# T cell–intrinsic VISTA expression promotes resistance to CTLA-4 blockade by restricting CD8^+^ T cell responses

**DOI:** 10.1172/JCI195668

**Published:** 2026-03-16

**Authors:** Cassandra Gilmour, Elizabeth DeLaney, Prerana B. Parthasarathy, Dia Roy, Hieu M. Ta, Amin Zakeri, Paolo Elguera Grandez, Sachin Patnaik, Keman Zhang, Ivan Juric, Rahul Rangan, Zahraa Al-Hilli, Anthony Tufaro, Booki Min, Samuel E. Weinberg, Timothy A. Chan, Natalie L. Silver, Stefanie Avril, Tyler Alban, Li Lily Wang

**Affiliations:** 1 Department of Translational Hematology & Oncology Research,; 2 Center for Immunotherapy & Precision Immuno-Oncology,; 3 Breast Center, Integrated Surgical Institute, and; 4 Plastic Surgery, Cleveland Clinic, Cleveland, Ohio, USA.; 5 Department of Microbiology and Immunology and; 6 Department of Pathology, Northwestern University Feinberg School of Medicine, Chicago, Illinois, USA.; 7 Department of Pathology, University Hospitals Cleveland Medical Center, Case Western Reserve University School of Medicine, Cleveland, Ohio, USA.; 8 Case Comprehensive Cancer Center, Cleveland, Ohio, USA.

**Keywords:** Immunology, Oncology, Adaptive immunity, Cancer immunotherapy, Cellular immune response

## Abstract

V-domain immunoglobulin suppressor of T cell activation (VISTA) is an immune checkpoint protein that impairs antitumor T cell responses. While broadly expressed on myeloid cells and T cells, the specific contribution of T cell–intrinsic VISTA to antitumor immunity remains undefined. This study investigated the phenotypic and functional consequences of T cell–specific VISTA deletion in tumor-specific CD8^+^ T cells. Single-cell transcriptomic analysis, TCR repertoire profiling, and flow cytometry revealed that loss of T cell–intrinsic VISTA enhanced early priming and short-term expansion of CD8^+^ T cells, yet this initial advantage failed to confer durable tumor control. Persistent dysfunction in VISTA-deficient T cells was in part driven by *trans*-VISTA on myeloid cells, while CTLA-4 upregulation further constrained T cell responses. T cell–intrinsic VISTA deficiency cooperated with CTLA-4 blockade to improve T cell survival and broaden TCR repertoire diversity, resulting in more robust tumor regression than CTLA-4 inhibition alone. A transcriptional signature enriched in VISTA-deficient cytotoxic T cells correlated with favorable outcomes in cancer patients treated with existing immune checkpoint inhibitors. These findings collectively define T cell–intrinsic mechanisms by which VISTA enforces T cell dysfunction and underscore its potential as both a therapeutic target and a biomarker of resistance to current immunotherapies.

## Abstract 2

This study reveals the role of T cell-intrinsic VISTA in modulating the responses of CD8+ cytotoxic T cells and promotes resistance to CTLA-4 targeted therapy.

## Graphical abstract

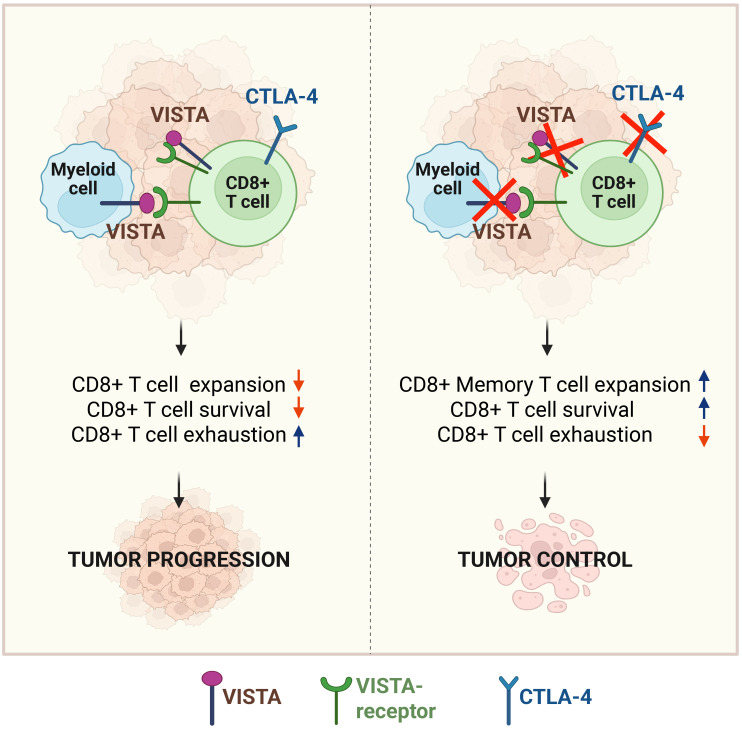

## Introduction

Immune checkpoint ligands and receptors, such as CTLA-4, PD-L1, and PD-1, represent a class of effective targets for cancer immunotherapy. Therapies using immune checkpoint inhibitors (ICIs) have improved survival in many late-stage cancer types (1–3). While the results are promising, the overall response rate to existing ICI therapies remains suboptimal due to various resistance mechanisms. One strategy to improve the therapeutic responses is to target alternative nonredundant immune checkpoint targets. V-domain immunoglobulin suppressor of T cell activation (VISTA), also known as Gi24, Dies-1, PD-1H, and DD1α, is an inhibitory B7 family immune checkpoint protein that suppresses antitumor immunity (4–6). VISTA is widely expressed on myeloid cells and T cells, where it plays multifaceted roles in modulating both myeloid cell– and T cell–mediated antitumor responses (4, 5, 7–15). Blocking VISTA, either by genetic deletion or using monoclonal antibodies, enhanced antitumor immunity in multiple preclinical tumor models (4, 12–15).

Among tumor-associated myeloid cells, VISTA is highly expressed on DCs, macrophages, and myeloid-derived suppressor cells (MDSCs). VISTA suppresses myeloid cell activation and polarization by inhibiting TLR signaling and reducing the production of cytokines and chemokines necessary for T cell recruitment (14). During tumor-driven myelopoiesis, VISTA expression on monocytic progenitors promotes their differentiation into MDSCs through enhancing STAT3 signaling (15). The T cell–suppressive function of VISTA is mediated through interactions with coinhibitory receptors (7, 16). LRIG1 is a newly identified coinhibitory receptor that binds to VISTA under both neutral pH and acidic pH conditions (16). This interaction dampens TCR signaling, limits T cell expansion and survival, and promotes T cell quiescence (16). In addition to LRIG1, PSGL-1 also engages VISTA specifically under acidic pH conditions (7). VISTA-specific antibodies with selective binding at acidic pH were found to accumulate within tumor tissues and inhibit tumor growth (7, 17).

Before the identification of LRIG1 and PSGL-1 as VISTA-interacting inhibitory receptors, VISTA was shown to be expressed on CD4^+^ T cells, where it intrinsically regulates their activation under neutral pH conditions (8) and helps to maintain the quiescence of naive CD4^+^ T cells (18). In contrast, the intrinsic role of VISTA in regulating CD8^+^ T cell responses has not been delineated. Our prior work demonstrated that VISTA expressed on T cells is functionally inert in the absence of LRIG1, whereas its *cis*-interaction with LRIG1 intrinsically suppresses TCR signaling (16). Based on this finding, we hypothesized that T cell–intrinsic VISTA may constrain the responses of antitumor CD8^+^ cytotoxic T cells (CTLs). To test this, we investigated antitumor immune responses in mice with T cell–specific VISTA deficiency. Our results indicate that although targeting T cell–intrinsic VISTA improved early T cell survival and accumulation, these effects were transient and insufficient to sustain a robust antitumor T cell response. T cell dysfunction was driven in part by *trans*-VISTA on myeloid cells and by CTLA-4 on CD8^+^ T cells. Combining T cell–intrinsic VISTA deficiency with CTLA-4 blockade overcame T cell dysfunction and promoted tumor regression. Mechanistic analyses elucidated the cooperative roles of T cell–intrinsic VISTA and CTLA-4 in regulating the survival, effector function, and TCR repertoire of tumor-specific CD8^+^ T cells. These findings support the future development of strategies to monitor and target T cell–intrinsic VISTA, which may help overcome therapeutic resistance to CTLA-4–directed therapies.

## Results

### VISTA expression was detected on murine and human CD8^+^ TILs.

**Figure 1:**
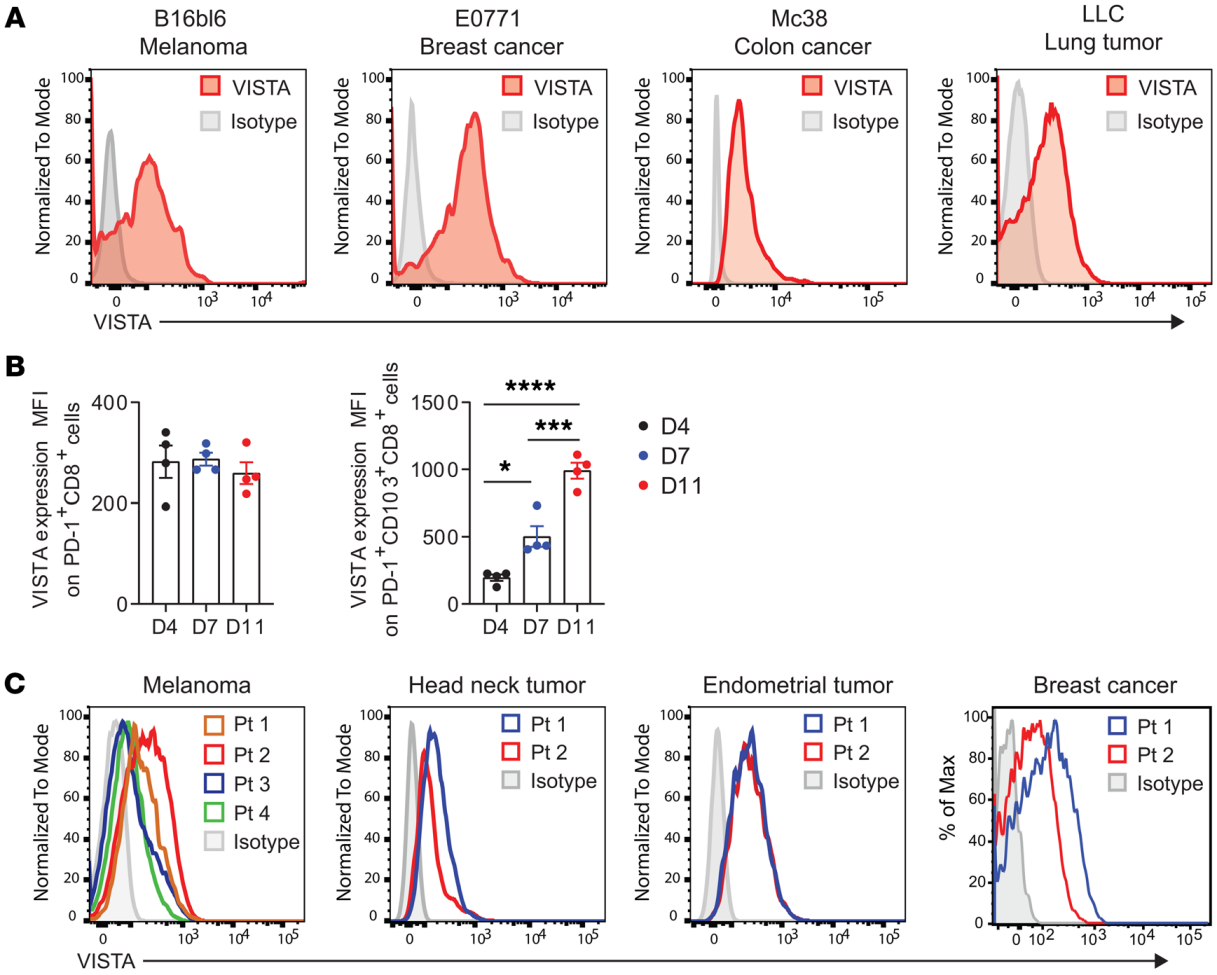
VISTA is abundantly expressed on CD8^+^ TILs in murine and human tumor tissues. (**A**) Mice were inoculated on the flank with murine tumors, including B16bl6 melanoma (100,000), E0771 breast cancer (100,000), MC38 colon cancer (100,000), and LLC lung cancer cells (100,000). Tumors were harvested when reaching diameters of approximately 7–8 mm, and TILs were examined for VISTA expression. At least 2 tumors were analyzed for each tumor type. Representative results are shown. (**B**) VISTA expression was quantified by MFI on CD8^+^ TILs from B16.OVA tumors, including the PD-1^+^ and PD-1^+^CD103^+^ subsets. *P < 0.05, ***P < 0.001, and ****P < 0.0001. Significance was determined by 1-way ANOVA. Data are presented as mean ± SEM. (**C**) VISTA expression on CD8^+^ TILs from surgically resected human tumor tissues was analyzed by flow cytometry. The data presented include CD8^+^ TILs from melanoma tissues, head and neck tumors, endometrial tumors, and breast cancer tissues. When available, multiple patient (Pt) specimens were included in the analysis.

We first analyzed the expression of VISTA on tumor-infiltrating lymphocytes (TILs) in murine tumor models and human tumor tissues. Murine tumor models such as B16bl6 melanoma, E0771 breast cancer, MC38 colon carcinoma, and Lewis lung carcinoma (LLC) tumors showed abundant VISTA expression on CD8^+^ TILs (Figure 1A). Expression of PD-1, 4-1BB, or CD103 may mark tumor-specific subsets within polyclonal CD8^+^ TIL populations (19–21). We observed that VISTA was coexpressed with these markers, as well as TIM3, on subsets of CTLs (Supplemental Figure 1; supplemental material available online with this article; https://doi.org/10.1172/JCI195668DS1). While VISTA expression on total PD-1^+^ CTLs remained stable, its expression on the PD1^+^CD103^+^ subset increased as tumors progressed (Figure 1B). VISTA expression was also detected on CD8^+^ TILs from human tumor tissues, including melanoma, head and neck cancer, endometrial cancer, and triple-negative breast cancer (Figure 1C). These results collectively indicate that VISTA is highly expressed on CD8^+^ TILs in solid tumor tissues and may modulate the functional responses of tumor-specific T cells.

### Deletion of T cell–intrinsic VISTA augmented the early responses of tumor-specific T cells.

**Figure 2:**
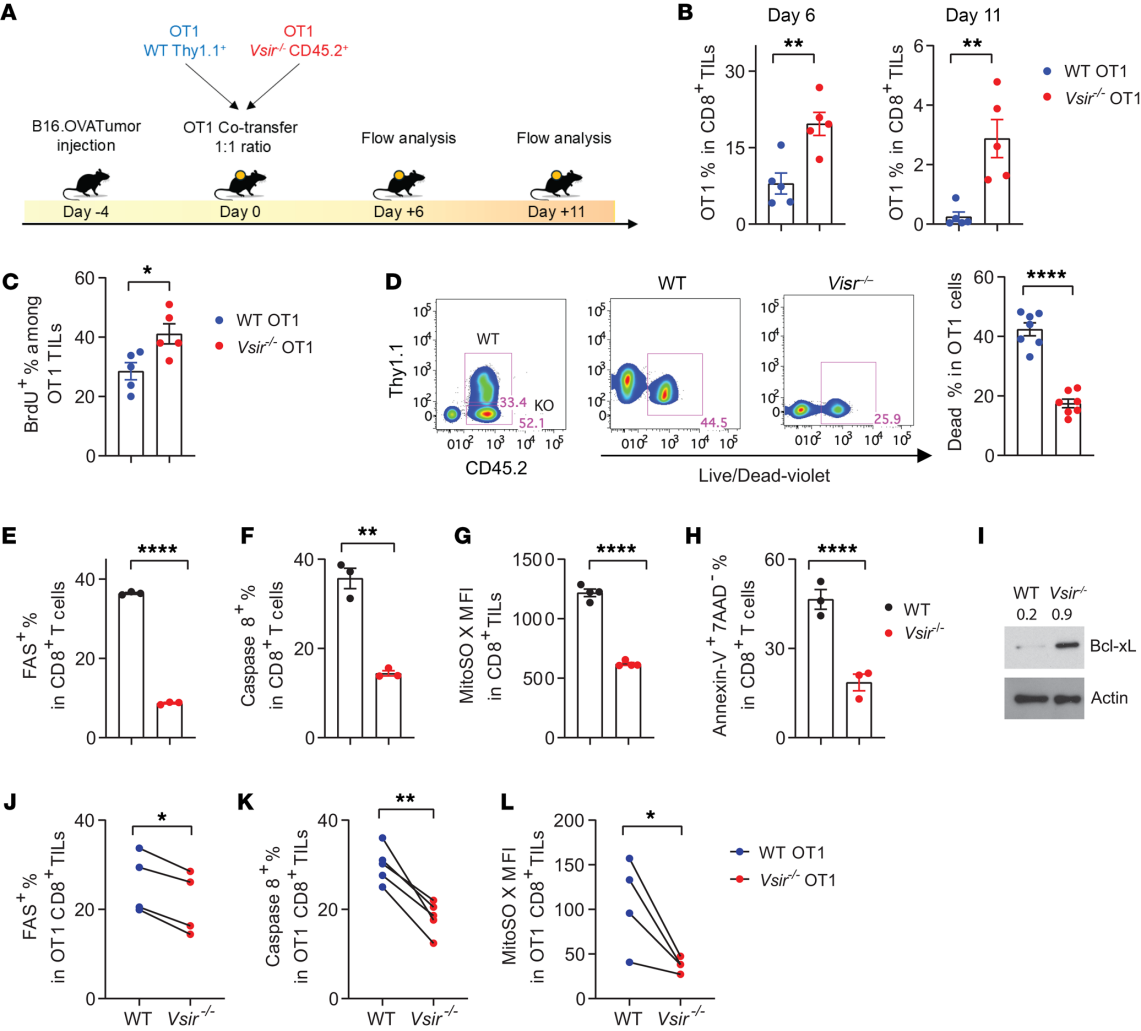
T cell–intrinsic VISTA deficiency enhanced the survival and accumulation of tumor-specific T cells in tumor tissues. Congenically marked WT OT1 (Thy1.1^+^) and *Vsir^–/–^* OT1 (CD45.2^+^) were mixed at a 1:1 ratio and adoptively cotransferred into C57bl/6 mice bearing 4-day established B16.OVA tumors. On days 6 and 11 after transfer, tumors were harvested, and the tumor-infiltrating OT1 T cells were examined. (**A**) Outline of the experimental design. (**B**) Percentages of WT and *Vsir^–/–^* OT1 T cells among total CD8^+^ TILs on days 6 and 11 after transfer. (**C**) Percentages of dead cells in WT and *Vsir^–/–^* OT1 TILs. (**D**) Mice were treated with BrdU 16 hours before tissue harvest and flow cytometry analysis. Percentages of BrdU^+^ OT1 T cells are shown. (**E**–**I**) WT and *Vsir^–/–^* CD8^+^ T cells were activated in vitro with anti-CD3 (3 mg/mL) for 48 hours before analysis. Shown are the expression levels of FAS and caspase-8 (**E** and **F**), mitochondrial ROS levels (**G**), and the frequencies of early apoptotic cells (annexin V^+^ 7AAD^–^) (**H**). Total cell lysates were collected and analyzed by Western blotting. Expression of Bcl-xL and β-actin is shown in **I**. (**J**–**L**) WT and *Vsir^–/–^* OT1 T cells were mixed and cotransferred into mice bearing B16.OVA tumors as in **A**. On day 10 after transfer, tumor-infiltrating OT1 T cells were analyzed for FAS (**J**) and caspase-8 (**K**) expression and mitoSOX levels (**L**). For **B** and **C**, *N* = 5. For **D**, *N* = 7. For **E**–**G**, and **I**, *N* = 4. For **J**–**L**, *N* = 4. *P < 0.05, **P < 0.01, ****P < 0.0001. Significance was determined by unpaired 2-tailed Student’s *t* test in all panels. Data are presented as mean ± SEM.

The inhibitory effects of VISTA on proximal TCR signaling have been attributed to its *cis*-interaction with LRIG1, a coinhibitory receptor (16). However, the impact of T cell–intrinsic VISTA on antitumor T cell responses remains unclear. To address this question, we generated VISTA-KO OT1 CD8^+^ TCR transgenic mice on a CD45.2 background (*Vsir^–/–^* OT1.CD45.2) and WT OT1 mice expressing the Thy1.1 congenic marker. To interrogate the behavior of OT1 T cells in response to tumor growth, we adoptively transferred *Vsir^–/–^* OT1.CD45.2 OT1 T cells together with WT.Thy1.1.OT1 T cells at a 1:1 ratio (5,000 each) into CD45.1 congenic hosts bearing established B16.OVA tumors (Figure 2A). This cotransfer approach permits WT and *Vsir^–/–^* OT1 T cells to coexist within the same tumor microenvironment (TME) while exhibiting distinct responses to the tumor, eliminating biases due to tumor size or structure. On day 6 after transfer, *Vsir^–/–^* OT1 T cells had accumulated to higher numbers than WT cells in the tumor tissues (Figure 2B). On day 11 after transfer, VISTA-KO OT1 T cells persisted in higher abundance than WT cells, although both populations declined (Figure 2B). *Vsir^–/–^* OT1 T cells exhibited enhanced proliferation, as indicated by increased BrdU incorporation (Figure 2C) and reduced cell death (Figure 2D). In addition to tumor tissues, *Vsir^–/–^* OT1 T cells also showed superior survival and accumulation in the tumor-draining lymph node (Supplemental Figure 2), indicating that the accumulation of *Vsir^–/–^* T cells within the tumor tissue was not due to elevated recruitment or retention. To investigate how VISTA regulates T cell survival, we analyzed apoptotic signaling in ex vivo activated T cells. Compared with WT T cells, *Vsir^–/–^* T cells displayed reduced expression of FAS and activated caspase-8, lower mitochondrial reactive oxygen species (ROS) levels (Figure 2, E–G, and gating strategies in Supplemental Figure 3), and increased Bcl-xL expression (Figure 2H). These changes were associated with decreased proportions of early apoptotic cells (annexin V^+^ 7AAD^–^) (Figure 2I). Moreover, we confirmed that *Vsir^–/–^* tumor-specific OT1 T cells exhibited reduced expression of FAS, diminished caspase-8 activation, and lower mitochondrial ROS levels within tumor tissues (Figure 2, J–L). These results suggest that VISTA promotes apoptosis in tumor-specific T cells.

To assess whether T cell–intrinsic VISTA regulates early antitumor T cell responses, WT and *Vsir^–/–^* OT1 cells were cotransferred into naive hosts 24 hours before tumor inoculation. By day 12 after transfer, VISTA-deficient OT1 cells showed greater accumulation in both the tumor-draining lymph node and tumor tissues compared with WT cells (Supplemental Figure 4, A and B). These results indicate that blocking T cell–intrinsic VISTA augments the initial response of tumor-specific CD8^+^ T cells by promoting their expansion and survival.

### T cell–intrinsic VISTA deficiency alone was insufficient to control tumor progression, owing to immunosuppression mediated by myeloid cell–derived VISTA.

**Figure 3:**
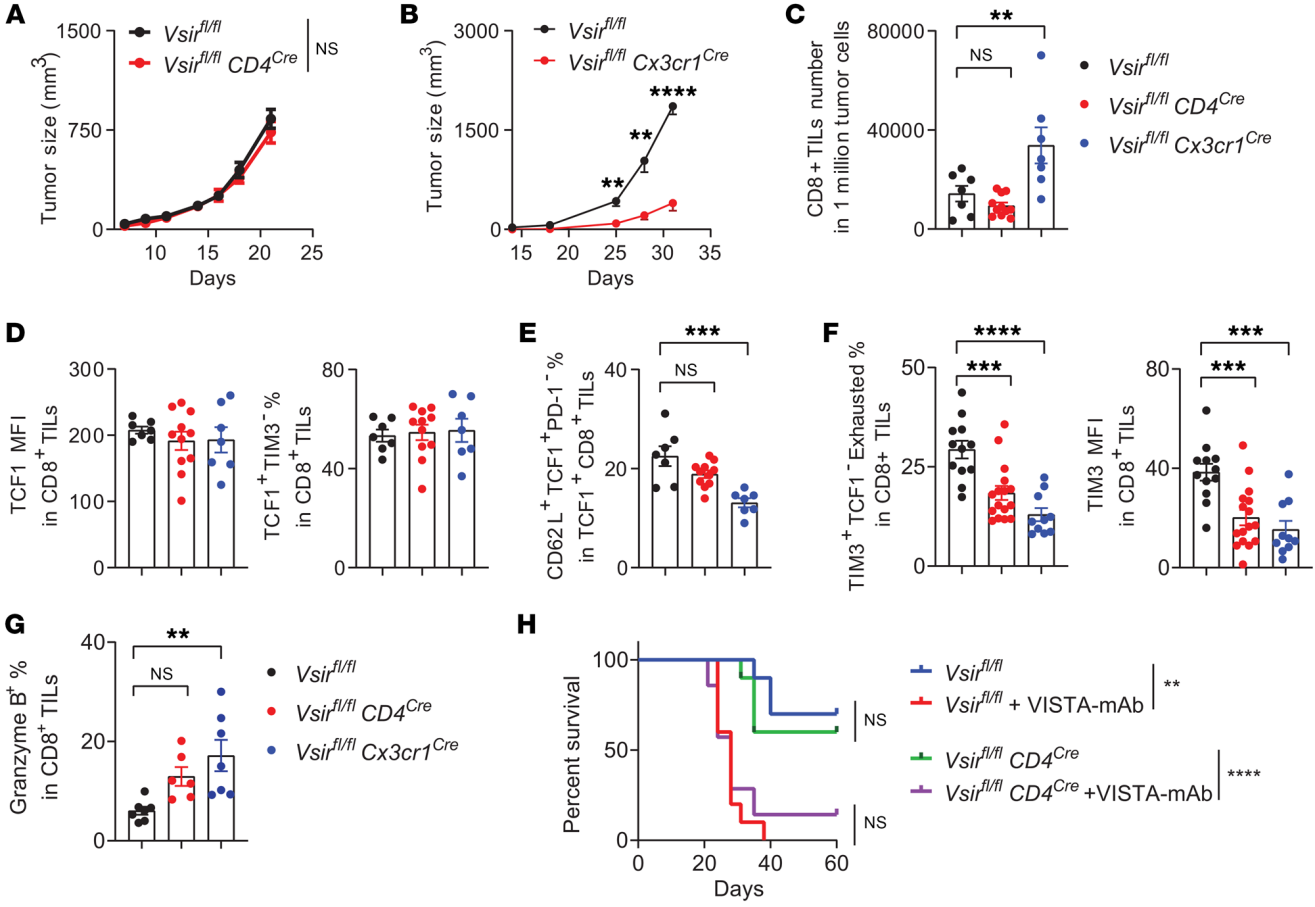
Myeloid-derived VISTA exerted a stronger suppressive effect on antitumor T cell responses than T cell–intrinsic VISTA. (**A** and **B**) T cell–specific VISTA-KO mice (*Vsir^fl/fl^ CD4^Cre^*) (**A**), myeloid-specific VISTA-KO mice (*Vsir^fl/fl^ Cx3cr1^Cre^*) (**B**), and WT controls (*Vsir^fl/fl^*) were inoculated with B16bl6 melanoma cells and treated with a TRP2 peptide vaccine on days 3 and 10. Tumor growths were monitored by a caliper. (**C**–**G**) WT and VISTA conditional KO mice as indicated were inoculated with B16bl6 melanoma cells and treated with 1 reduced dose of vaccine. Phenotypes of CD8^+^ TILs were examined by flow cytometry on days 14–16 after vaccine treatment. Normalized numbers of CD8^+^ TILs are shown in **C**. TCF1 expression levels, measured as MFI, and frequencies of TCF1^+^TIM3^–^ stem-like cells are shown in **D**. Frequencies of TCF1^+^CD62L^+^PD-1^–^ quiescent T cells are shown in **E**. Frequencies of TCF1^–^Tim3^+^ T cells and TIM3 expression levels are shown in **F**. Granzyme B expression is shown in **G**. (**H**) Mice were inoculated with B16bl6 melanoma and treated with peptide vaccine on days 3 and 10 or treated with anti-VISTA antibodies (200 mg per mouse) every 2–3 days. Mice survival up to 60 days after tumor inoculation is shown. Data are presented as mean ± SEM. For **A**, *N* = 10 (*Vsir^fl/fl^*) and 9 (*Vsir^fl/fl^* *CD4^Cre^*). For **B**, *N* = 10 (*Vsir^fl/fl^*) and 11 (*Vsir^fl/fl^* *Cx3cr1^Cre^*). For **C**–**E**, *N* = 7 (*Vsir^fl/fl^*), 11 (*Vsir^fl/fl^* *CD4^Cre^*), and 7 (*Vsir^fl/fl^* *Cx3cr1^Cre^*). For **F**, *N* = 12 (*Vsir^fl/fl^*), 16 (*Vsir^fl/fl^* *CD4^Cre^*), and 10 (*Vsir^fl/fl^* *Cx3cr1^Cre^*). For **G**, *N* = 7 (*Vsir^fl/fl^*), 6 (*Vsir^fl/fl^* *CD4^Cre^*), and 7 (*Vsir^fl/fl^* *Cx3cr1^Cre^*). For **H**, *N* = 7 (*Vsir^fl/fl^*), 10 (*Vsir^fl/fl^*+mAb), 9 (*Vsir^fl/fl^* *CD4^Cre^*), and 10 (*Vsir^fl/fl^CD4^Cre^*+mAb). **P < 0.01, ***P < 0.001, ****P < 0.0001. Significance was determined using 2-way ANOVA with a Šidák’s multiple-comparison test (**A** and **B**), 1-way ANOVA (**C** and **E**–**G**), or log-rank Mantel-Cox test (**H**). All experiments were repeated 2–3 times.

To investigate how T cell–intrinsic VISTA controls tumor growth, we generated T cell–specific VISTA conditional KO mice by breeding *Vsir ^fl/fl^* mice to CD4^cre^ mice on the C57BL/6 background (15, 22). We have previously shown that the B16bl6 melanoma grew at similar rates in VISTA global KO mice and their WT littermates, likely due to the nonimmunogenic nature of this tumor model (11). Treatment with a peptide vaccine composed of TRP2-derived peptides together with TLR7/8/9 agonists CpG and R848 as adjuvants induced tumor regression in VISTA global KO mice (11). To assess the contribution of T cell–intrinsic VISTA to vaccine-induced antitumor immunity, T cell–specific VISTA conditional KO mice (*Vsir^fl/fl^* *CD4*^cre^) and WT (*Vsir^fl/fl^*) littermates bearing 3-day established B16l6 tumors were treated with the TRP2 peptide vaccine and monitored for tumor growth. Unexpectedly, vaccination did not affect tumor progression in T cell–intrinsic VISTA-KO mice (Figure 3A).

This finding contrasts with the robust tumor control seen in global VISTA-KO mice (11) and suggests the involvement of additional immunosuppressive mechanisms. Recent studies have shown that tumor-associated MDSCs, DCs, or macrophages express high levels of VISTA (14, 15). VISTA-deficient T cells remain sensitive to *trans*-VISTA–mediated suppression through LRIG1-dependent inhibitory signaling (Supplemental Figure 5) (16). We hypothesized that *trans*-VISTA expressed on tumor-associated myeloid cells promotes dysfunction of VISTA-deficient T cells. To evaluate the role of myeloid-derived VISTA, we examined the growth of B16bl6 tumors in myeloid-specific VISTA-KO mice (*Vsir ^fl/fl^* Cx3CR1^cre/+^). VISTA deficiency in myeloid cells inhibited tumor growth following peptide vaccination (Figure 3B). To determine the mechanisms of tumor control, we compared the phenotypes of CD8^+^ TILs in mice with T cell–intrinsic versus myeloid-specific VISTA deletion. We observed that tumor control in myeloid-specific VISTA-KO mice was associated with increased accumulation of CD8^+^ TILs (Figure 3C). While TCF1 expression and the frequency of TCF1^+^TIM3^–^CD8^+^ TILs remained unchanged (Figure 3D), CD8^+^ TILs within the myeloid-specific VISTA-deficient TME (*Vsir^fl/fl^ Cx3cr1^Cre^*) exhibited reduced frequencies of quiescent (TCF1^+^CD62L^+^PD-1^–^) (Figure 3E) and exhausted (TCF1^–^TIM3^+^) phenotypic subsets (Figure 3F), accompanied by increased granzyme B expression (Figure 3G). In contrast, deletion of T cell–intrinsic VISTA did not significantly reduce the quiescent subset or increase granzyme B expression, although it did reduce TIM3 expression (Figure 3, E–G). Anti-VISTA antibody treatment suppressed tumor growth in T cell–intrinsic VISTA-KO mice as effectively as global VISTA blockade in WT mice (Figure 3H). Taken together, these results indicate that VISTA-deficient T cells remain susceptible to myeloid-derived *trans*-VISTA, which plays a dominant role in enforcing T cell quiescence and limiting their cytotoxic function.

### CTLA-4 blockade synergized with T cell–intrinsic VISTA deficiency enhanced antitumor T cell responses.

**Figure 4:**
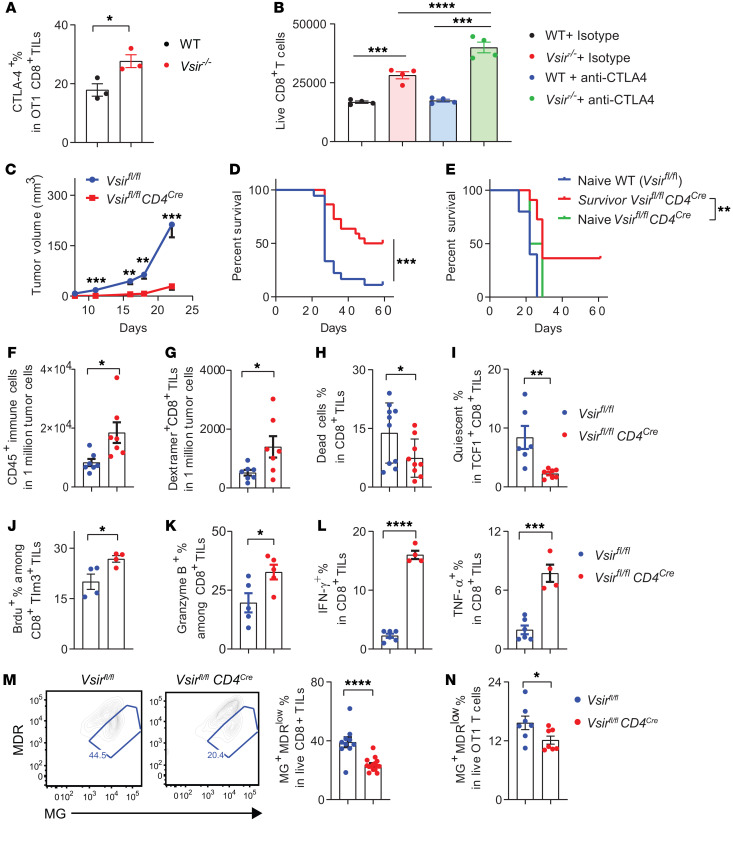
T cell–intrinsic VISTA deficiency synergized with CTLA-4 blockade to boost antitumor T cell responses. (**A**) CTLA-4 expression on tumor-infiltrating OT1 T cells adoptively transferred into mice bearing B16.OVA tumors. *N* = 3. (**B**) WT and *Vsir^–/–^* CD8^+^ T cells were activated with anti-CD3 in the presence of anti–CTLA-4 antibody or isotype IgG. Expanded live T cells after 72 hours were enumerated. *N* = 4. (**C** and **D**) VISTA-KO mice (*Vsir^fl/fl^ CD4^Cre^*, *N* = 22) and WT controls (*N* = 18) bearing B16bl6 tumors were treated with a peptide vaccine and anti–CTLA-4 antibodies. Tumor progression is shown in **C**. Mice survival up to 60 days is shown in **D**. (**E**) Tumor-free mice were rechallenged with melanoma cells and monitored for tumor growth. *N* = 5 (naive WT), 11 (tumor-free *Vsir^fl/fl^* *CD4^Cre^*), and 4 (naive *Vsir^fl/fl^* *CD4^Cre^*). (**F**–**M**) CD8^+^ TILs were analyzed on day 16 after treatment with the vaccine and anti-CTLA4 antibodies. (**F**) Numbers of CD45^+^ TILs. (**G**) Frequencies of CD8^+^ TILs recognized by TRP2-specific dextramer. *N* = 7 for **F** and **G**. (**H**) Frequencies of dead CD8^+^ TILs. *N* = 10 (*Vsir^fl/fl^*), 9 (*Vsir^fl/fl^* *CD4^Cre^*). (**I**) Frequencies of quiescent CD8^+^ TILs (TCF1^+^CD62L^+^PD-1^–^). *N* = 6. (**J**) Frequencies of BrdU-incorporating CD8^+^ TILs. *N* = 4. (**K**) Expression of granzyme B (*N* = 5). (**L**) Expression of IFN-γ and TNF-α. *N* = 6 (*Vsir^fl/fl^*) and 4 (*Vsir^fl/fl^* *CD4^Cre^*). (**M**) Representative flow plots of CD8^+^ TILs stained with MG and MDR dyes and frequencies of MG^+^MDR^lo^ cells. (**N**) WT and *Vsir^–/–^* OT1 T cells (*N* = 7) were cotransferred at a 1:1 ratio into mice bearing B16.OVA tumors and analyzed after 9 days. Frequencies of MG^+^MDR^lo^ OT1 TILs are shown. For **M**, *N* = 10 (*Vsir^fl/fl^*) and 15 (*Vsir^fl/fl^* *CD4^Cre^*). *P < 0.05, **P < 0.01, ***P < 0.001, ****P < 0.0001. Significance was assessed using unpaired 2-tailed Student’s *t* test (**A** and **F**–**N**), 1-way ANOVA (**B**), 2-way ANOVA with a Šidák’s multiple-comparison test (**C**), or log-rank Mantel-Cox test (**D** and **E**). Data are presented as mean ± SEM. All experiments were repeated 2–3 times.

Given the unrestrained tumor progression seen in T cell–intrinsic VISTA-KO mice, we hypothesized that additional immune checkpoint receptors may contribute to the dysfunction of VISTA-deficient T cells. An earlier study showed that VISTA-blocking antibodies synergized with CTLA-4 inhibitors to control tumor growth in a preclinical model of squamous cell carcinoma (23). We found that *Vsir^–/–^* CTLs expressed higher levels of CTLA-4 than WT CD8^+^ T cells, both in vitro (Supplemental Figure 6) and in vivo (Figure 4A). Moreover, treatment with CTLA-4 blocking antibody synergized with VISTA deficiency to optimally expand T cells (Figure 4B). These results prompted us to evaluate the effects of CTLA-4 blockade in WT and *Vsir^fl/fl^* CD4^cre^ mice bearing B16 melanoma. Treatment with a peptide vaccine combined with anti–CTLA-4 antibodies effectively inhibited tumor progression in *Vsir ^fl/fl^* CD4^cre^ mice, but not in WT controls (Figure 4C). A substantial portion of VISTA-KO mice achieved tumor-free survival for at least 60 days, whereas all WT mice succumbed to tumor burden (Figure 4D). To assess polyclonal T cells in tumor-free mice, we analyzed CD8^+^ T cell phenotypes across spleen, lymph nodes, liver, BM, and blood at day 60 after tumor regression. T cell abundance and activation status, as defined by CD44 and CD62L expression, were comparable between tumor-free mice and naive controls (Supplemental Figure 7, A–C), indicating normal baseline T cell homeostasis after tumor clearance. TRP2-specific CD8^+^ T cells, identified using a TRP2-specific dextramer, were more enriched in the BM and liver of tumor-free mice (Supplemental Figure 7D). To assess their effector function, we stimulated T cells ex vivo with TRP2 peptides. Compared with naive controls, BM CD8^+^ T cells from tumor-free VISTA-KO mice showed increased expression of CD107a and TNF-α (Supplemental Figure 7, E and F), consistent with the generation of TRP2-specific memory CD8^+^ T cells following tumor clearance.

To evaluate the protective capacity of memory responses, tumor-free mice were rechallenged with tumors on day 60 after the initial treatment. Naive WT mice and *Vsir ^fl/fl^* CD4^cre^ mice were analyzed as parallel controls. A significant fraction of tumor-free VISTA-KO survivor mice was resistant to tumor rechallenge (Figure 4E), indicating protection mediated by tumor-specific memory T cell responses. Similar results were seen in the MC38 colon cancer model, where combined CTLA-4 blockade and peptide vaccination led to tumor-free survival in T cell–intrinsic VISTA-KO mice, which also resisted tumor rechallenge (Supplemental Figure 8).

To understand the mechanisms of tumor control in T cell–intrinsic VISTA-KO mice, we investigated the abundance, phenotypes, and effector function of CD8^+^ TILs by flow cytometry. Higher numbers of total CD45^+^ tumor-infiltrating immune cells were seen in VISTA-deficient mice (Figure 4F). Using TRP2-specific dextramer, we detected a higher abundance of tumor-specific *Vsir^–/–^* CD8^+^ TILs than WT cells (Figure 4G). Furthermore, *Vsir^–/–^* CD8^+^ TILs exhibited reduced death (Figure 4H) and a decreased proportion of quiescent T cells (TCF1^+^CD62L^+^PD-1^–^) (Figure 4I). TIM3^+^ *Vsir^–/–^* CD8^+^ TILs were more proliferative than WT counterparts, as indicated by increased BrdU incorporation (Figure 4J). *Vsir^–/–^* CD8^+^ TILs exhibited enhanced effector activity, with elevated expression of granzyme B (Figure 4K) and effector cytokines IFN-γ and TNF-α (Figure 4L). Taken together, these results indicate that in conjunction with CTLA-4 blockade, loss of T cell–intrinsic VISTA enhanced the proliferation, survival, and effector function of tumor-specific CD8^+^ T cells.

We next explored the mechanisms by which T cell–intrinsic VISTA regulates the survival and function of tumor-specific T cells in the context of CTLA-4 blockade. We hypothesized that the augmented proliferative capacity and effector function of *Vsir^–/–^* CD8^+^ T cells may result from metabolic adaptations, as previous studies have implicated dysfunctional mitochondria as early drivers of T cell dysfunction (24, 25). We examined mitochondrial status in polyclonal CD8^+^ TILs using the mitochondria-specific dyes MitoTracker Green FM (MG) and MitoStain Deep Red (MDR) that detect mitochondrial mass and membrane polarization, respectively (26–28). Our results revealed that VISTA-deficient CD8^+^ TILs contained a reduced proportion of MG^hi^MDR^lo^ cells carrying depolarized mitochondria (Figure 4M). To confirm this observation in tumor-specific CD8^+^ T cells, we analyzed mitochondria in WT versus *Vsir^–/–^* OT1 T cells cotransferred into B16.OVA tumor-bearing mice and observed similar results (Figure 4N). To investigate the role of T cell–intrinsic VISTA in modulating mitochondrial bioenergetics, we performed a Seahorse XF Mito stress test to assess the oxidative consumption rate (OCR) of in vitro–cultured OT1 T cells. Forty-eight hours after anti-CD3/CD28 stimulation, *Vsir^–/–^* T cells exhibited a modest increase in basal respiration and a significantly enhanced spare reserve capacity (SRC) (Supplemental Figure 9A). Since repeated TCR stimulation drives T cell dysfunction (24), we examined mitochondrial respiration after a second round of TCR stimulation. *Vsir^–/–^* T cells maintained superior mitochondrial SRC compared with WT T cells (Supplemental Figure 9B). These findings corroborate with elevated mitochondrial membrane potential in *Vsir^–/–^* CD8^+^ TILs and indicate that T cell–intrinsic VISTA acts to impair mitochondrial bioenergetics and metabolic fitness of CD8^+^ T cells.

### Single-cell profiling revealed that T cell–intrinsic VISTA deficiency expanded the effector memory subset and increased the TCR diversity in CD8^+^ TILs.

**Figure 5:**
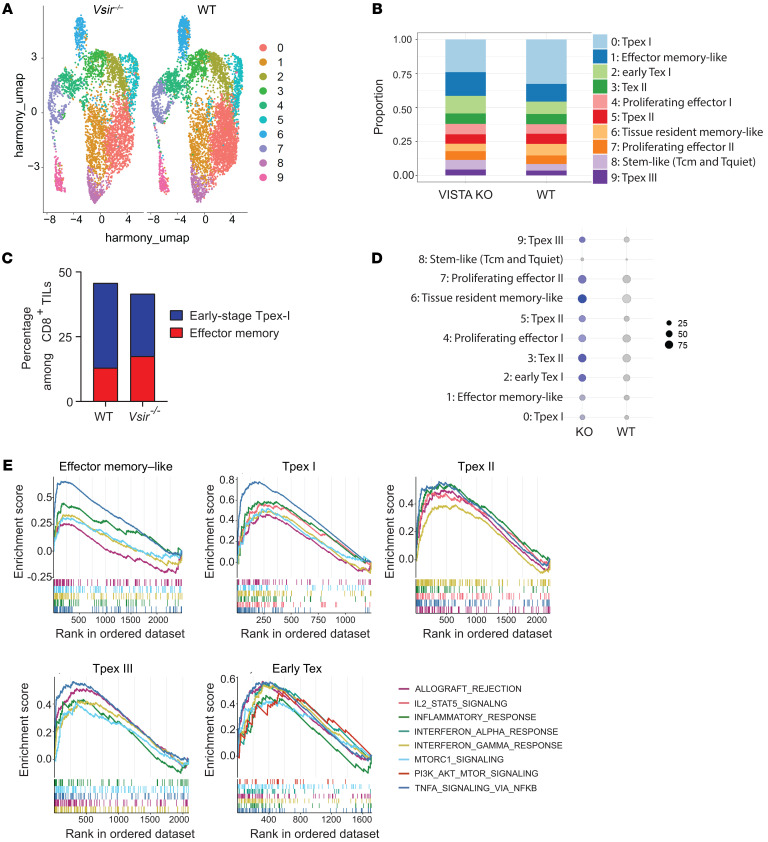
Single-cell transcriptome analysis revealed the molecular pathways in CD8^+^ T cells that were regulated by T cell–intrinsic VISTA. T cell–specific VISTA-KO mice (*Vsir^fl/fl^ CD4^Cre^*) and WT littermates bearing B16 melanoma were treated with a combination of peptide vaccine and anti–CTLA-4 antibodies. On day 18 after treatment, CD8^+^ TILs were isolated from pooled tumor tissues (6–8 tumors for each genotype) and analyzed by scRNA-seq. (**A**) UMAP clusters of CD8^+^ TIL subsets. (**B**) The identities and proportions of WT and *Vsir^–/–^* CD8^+^ TIL subsets. (**C**) The proportions of the early-stage Tpex-I and effector memory Tem subsets in WT versus *Vsir^–/–^* CD8^+^ TILs. (**D**) *Ctla4* gene expression in WT and *Vsir^–/–^* CD8^+^ TIL subsets. (**E**) GSEA leading-edge analysis of CD8^+^ TILs was performed to identify molecular pathways enriched in *Vsir^–/–^* CD8^+^ TILs. Shared molecular pathways enriched in several *Vsir^–/–^* CD8^+^ TIL subsets are shown.

Previous studies have determined that tumor-associated lymphocytes are heterogeneous and that ICI therapies elicit measurable alterations in T cell repertoire, differentiation, and functional states (29–34). To understand the heterogeneity of CD8^+^ T cells, we isolated WT and *Vsir^–/–^* CD8^+^ TILs and performed single-cell transcriptomic profiling coupled with single-cell TCR repertoire analysis. After quality control, we identified 5,861 WT and 4,555 *Vsir^–/–^* CD8^+^ TILs. Unsupervised clustering gave rise to 10 distinct clusters (Figure 5A). Cluster identities were defined based on markers associated with T cell activation and functional states (35) (Figure 5B and Supplemental Figure 10). Multiple clusters (C0, C1, C5, C6, C8, and C9) expressed *Tcf7*, a gene associated with T cell stemness (36, 37). Both C1 and C6 lacked *Sell* and *Ccr7* expression but expressed other memory-like genes (e.g., *Tcf7*, *Il7r*, *Klf2*, *Lef1*, *S1pr1*, and *Slamf6*) and activation/effector function genes (e.g., *Klrk1*, *Cd44*, *Cd69*, *Ifng*, *Tnf*, *Gzmb*, and *Prf1*). C6 also expressed Itgae, the marker for tissue resident memory (Trm) cells (38). Thus, C1 resembled effector memory-like (Tem) cells, and C6 resembled Trm cells. Compared with C1, C0/C5/C9 expressed lower levels of *Tcf7* but lost several memory/stem-like markers (e.g., *Il7r*, *Ccr7*, and *Lef1*). They expressed effector function genes (*Ifng*, *Tnf*, *Gzmb*, and *Prf1*) and intermediate levels of inhibitory receptors (e.g., *Pdcd1* and *Tigit*) but not *Havcr2*. C5/C9 also expressed *Lag3* and *CTLA-4*, as well as transcription factors *Nr4a2* and *Tox*. Thus, C0/C5/C9 clusters represent progenitor exhausted (Tpex) T cells at distinct stages of differentiation, with C0 as earlier stage Tpex than C5/C9. C8 expressed high levels of naive/stem-like gene signatures (e.g., *Tcf7*, *Il7r*, *Sell*, *Ccr7*, *Klf2*, *Lef1*, *S1pr1*, and *Slamf6*) but lacked expression of inhibitory receptors and effector function genes. The majority of C8 cells expressed activation marker *Cd69* and resembled central memory-like cells. A fraction of C8 cells were negative for *Cd69*, resembling the quiescent naive-like subset. The remaining clusters C2/C3/C4/C7 lacked *Tcf7* expression, consistent with a terminally differentiated phenotype. C4/C7 expressed Mki67, indicating their proliferative states (proliferating effector cells). C2 and C3 were not proliferative based on the lack of Mki67 expression and expressed inhibitory receptors (e.g., *Pdcd1*, *Lag3*, *Harvcr2*, *Tigit*, and *CTLA-4*) and transcription factors (*Nr4a2* and *Tox*). Thus, C2/C3 represented terminally exhausted (Tex) subsets, although C2 appeared less exhausted, exhibiting lower expression of Havcr2 and Cd101 (39).

Quantification of clusters revealed that VISTA-deficient CD8^+^ TILs contained a higher abundance of effector memory subset (C1, from 12.9% in WT T cells to 17.5% in VISTA-KO T cells) and early Tex subset (C2, from 9.1% in WT T cells to 12.9% in VISTA-KO T cells). In contrast, VISTA deficiency reduced the frequency of the early-stage Tpex-I subset (C0, from 32.76% in WT T cells to 24.08% in VISTA-KO T cells) (Figure 5, B and C). These findings suggest that VISTA deficiency promotes the differentiation of CD8^+^ T cells toward effector memory and less exhausted phenotypes, contributing to more sustained tumor control.

To investigate the molecular programs regulated by VISTA, we analyzed differentially expressed genes in CD8^+^ TILs. Notably, *Ctla4* gene expression was upregulated across multiple subsets of VISTA-deficient CD8^+^ TILs (Figure 5D), consistent with findings in in vitro–activated T cells (Supplemental Figure 6) and in vivo OT1 CD8^+^ TILs (Figure 4A). Next, we performed GSEA to elucidate VISTA-dependent molecular pathways (Figure 5E). Volcano plots display representative profiles of differential gene expression in Tpex-I and Tem subsets (Supplemental Figure 11). Several pathways, such as allograft rejection, IL-2/STAT5 signaling, inflammatory response, IFN-α and IFN-γ response, mTORC1 signaling, AKT_MTOR signaling, and TNF-α signaling via NF-κB were upregulated in multiple subsets of *Vsir^–/–^* CD8^+^ TILs, including the Tpex populations (C0/C5/C9), Tem (C1), and early Tex (C2) subsets. IL-2/STAT5 signaling is known to antagonize the TOX-mediated T cell exhaustion program, while MTOR signaling promotes cell growth, metabolism, and survival (40–42). These pathway alterations indicate that T cell–intrinsic VISTA regulates the expansion, survival, and effector function of tumor-specific CD8^+^ T cells.

**Figure 6:**
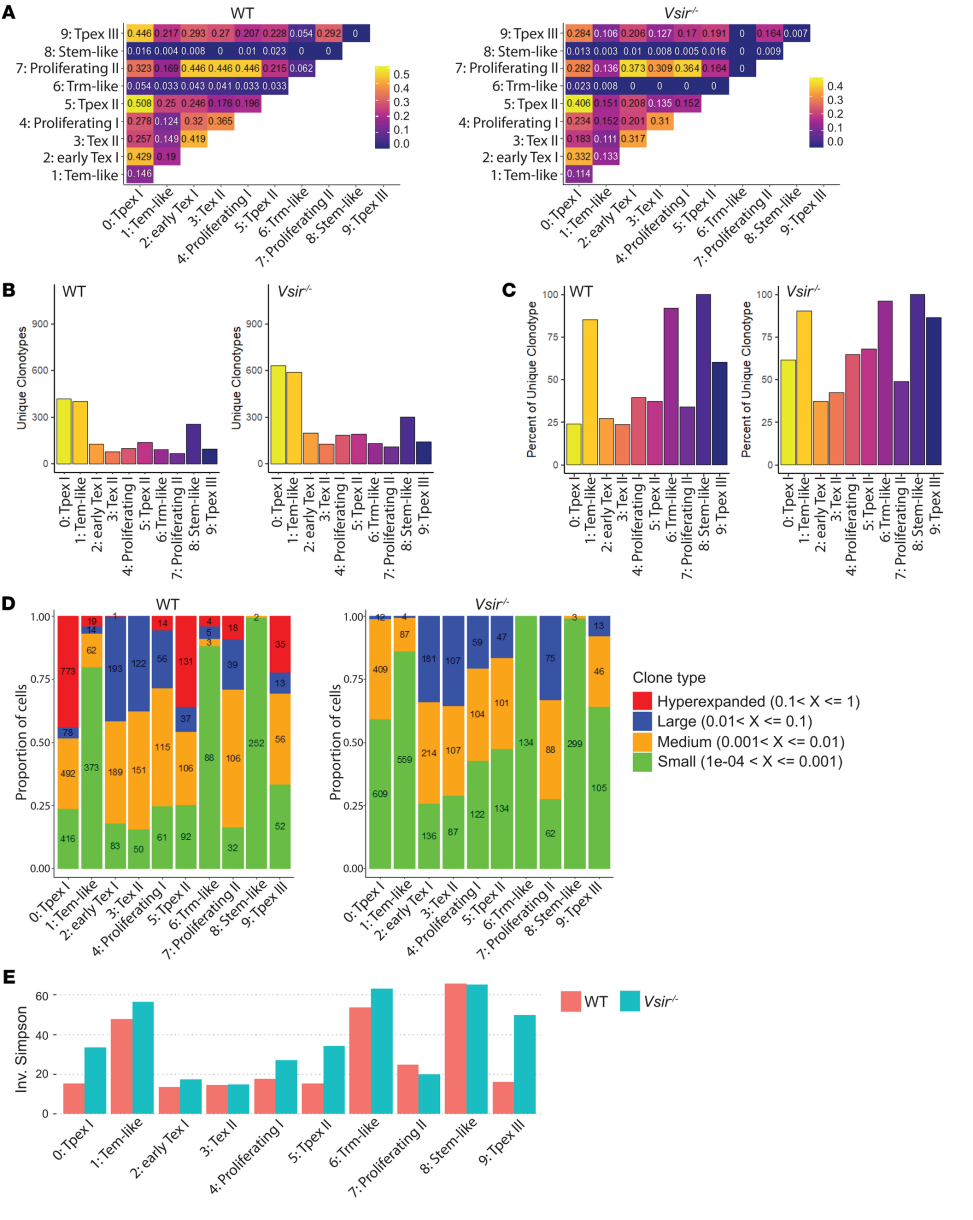
Single-cell TCR analysis revealed increased TCR repertoire diversity in VISTA-deficient CD8^+^ TILs. The TCR repertoire of WT and VISTA-deficient CD8^+^ TILs was sequenced and analyzed. (**A**) The extent of clonotype overlap between CTL subsets was quantified. (**B**) The number of unique TCR clonotypes was quantified within each CTL subset. (**C**) The percentages of cells harboring unique TCR clonotypes are displayed for each CTL subset. (**D**) TCR clonotypes were classified based on their expansion levels into hyperexpanded, large, medium, and small categories, and their distribution across each CTL subset is presented. (**E**) The overall TCR repertoire diversity was quantified using the inverse Simpson index and presented for each T cell subset.

Beyond effects on gene expression, we postulated that VISTA deficiency may modulate the TCR repertoire, thereby promoting more effective antitumor responses. This hypothesis is supported by studies showing that greater TCR repertoire diversity correlates with improved clinical responses to CTLA-4 blockade (29–34). To formally address this hypothesis, we examined single-cell TCR sequences in the context of transcriptionally defined CD8^+^ TIL subsets. Analysis of overlapping clonotypes revealed that both WT and *Vsir^–/–^* CD8^+^ TILs showed the highest degree of overlap among the Tpex subsets C0, C5, and C9, suggesting a closely related differentiation relationship between these populations (Figure 6A). The proliferating subsets C4 and C7 shared high levels of overlapping clonotypes with each other and with the Tpex C0 and the Tex C2/C3 subsets, indicating their interconnected differentiation relationship. The Tem C1 exhibited lower clonotype overlaps with other subsets, indicating that Tem cells follow a distinct differentiation trajectory (Figure 6A). Moreover, the degrees of clonotype overlap between all subsets were consistently lower in *Vsir^–/–^* CD8^+^ T cells than in WT T cells (Figure 6A). Quantification of the number and frequency of unique clonotypes revealed that *Vsir^–/–^* CD8^+^ T cells harbored a greater number of unique clonotypes across all subsets (Figure 6, B and C) and contained fewer hyperexpanded clonotypes (Figure 6D), thereby exhibiting greater TCR repertoire diversity based on the inverse Simpson index (Figure 6E). These findings suggest that T cell–intrinsic VISTA deficiency enhances clonotype diversity, which contributes to durable tumor control in combination with CTLA-4 blockade.

### A gene signature enriched in VISTA-deficient CD8^+^ T cells is associated with improved clinical responses to immunotherapy.

Our transcriptomic analysis revealed reprogramming of gene expression in VISTA-deficient T cells. Since VISTA is expressed on CD8^+^ TILs in human tumors, we investigated the prognostic role of T cell–intrinsic VISTA in human melanoma. We identified the top 40 differentially expressed genes enriched in murine *Vsir^–/–^* CD8^+^ TILs and mapped them to their human orthologs (Supplemental Figure 12, A and B). This gene module reflects the functional profile of CD8^+^ CTLs regulated by VISTA and thus designated the VISTA^deficient^ CTL signature. Using a public scRNA-seq dataset of pan-cancer tissues (43), we confirmed that this signature is most strongly enriched in tumor-infiltrating T cells compared with malignant cells and other immune populations (Supplemental Figure 12C). We evaluated the association between this signature and clinical responses in patients with triple-negative breast cancer treated with chemotherapies and PD-L1 blockade (44). The VISTA^deficient^ CTL signature was significantly enriched in pretreatment CD8^+^ TILs from responders compared with nonresponders (*P* = 0.047), supporting its prognostic value (Supplemental Figure 12D).

**Figure 7:**
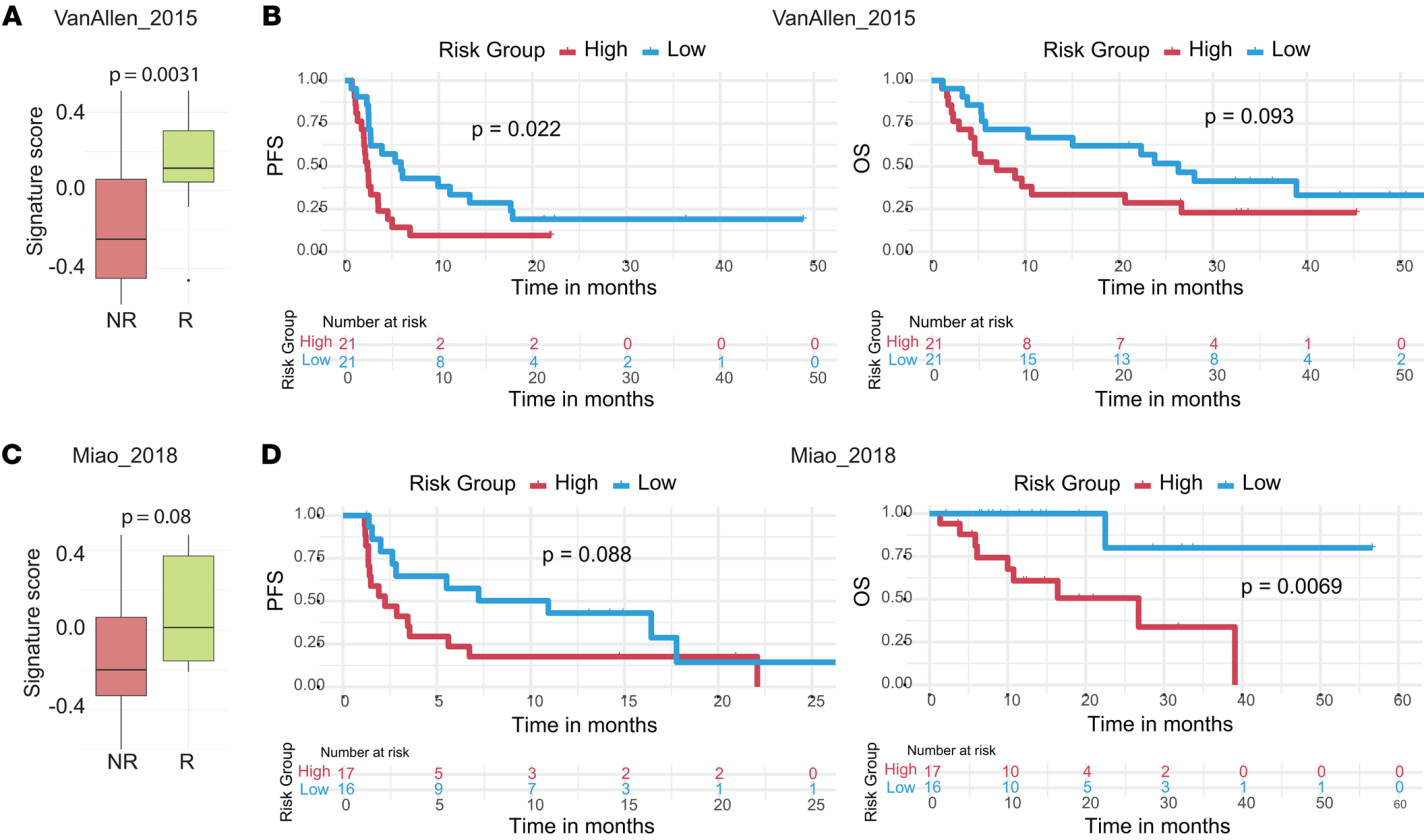
The prognostic role of the VISTA^deficient^ CTL gene signature in human cancers treated with ICI. Bulk RNA-seq datasets, including the melanoma study by Van Allen et al. (46) (**A** and **B**) and the clear-cell renal cell carcinoma study by Miao et al. (47) (**C** and **D**), were analyzed. Expression of the VISTA^deficient^ CTL signature is shown in **A** and **C**. The association between the signature score and survival outcomes, including PFS and OS, is shown in **B** and **D**. Significance was determined using unpaired 2-tailed Student’s *t* test (**A** and **C**) and log-rank Mantel-Cox test (**B** and **D**).

Since CTLA-4 is upregulated in murine VISTA-deficient T cells (Figure 4A and Figure 5D), we analyzed *Ctla4* expression in human CD8^+^ TILs using a public scRNA-seq dataset from melanoma tumors (45). *Ctla4* expression was elevated in T cells with higher signature scores (Supplemental Figure 13). Next, we examined the association of the VISTA^deficient^ CTL signature with clinical responses in melanoma patients treated with ipilimumab (46). In pretreatment tissues, the signature score was significantly higher in responders than in nonresponders (*P* = 0.0031) (Figure 7A) and was associated with improved progression-free survival (PFS; *P* = 0.022), while showing a trend toward improved overall survival (OS; *P* = 0.093) (Figure 7B). Lastly, we evaluated the prognostic relevance of the CTL signature in patients with metastatic clear-cell renal cell carcinoma treated with PD-1 and/or CTLA-4 inhibitors (47). The VISTA^deficient^ CTL signature was more enriched in responders than in nonresponders (Figure 7C), correlated with improved OS (*P* = 0.0069), and showed a trend toward better PFS (*P* = 0.088) (Figure 7D). These results collectively indicate that T cell–intrinsic VISTA plays a key role in restraining antitumor CD8^+^ T cell responses and diminishing clinical efficacy of immunotherapies targeting CTLA-4 and PD-L1/PD-1.

## Discussion

Previous studies have suggested that VISTA expressed on T cells intrinsically modulates T cell activation. For example, an earlier study by Flies et al. demonstrated that VISTA-deficient CD4^+^ T cells exhibit enhanced proliferation and cytokine production (8). Similarly, work by Ta et al. revealed that VISTA deletion in CD8^+^ T cells augments TCR signaling and T cell activation, primarily through a *cis*-interaction with the coinhibitory receptor LRIG1 (16). These initial findings raise important questions about the role of T cell–intrinsic VISTA in shaping the responses of tumor-specific CD8^+^ T cells and contributing to the therapeutic resistance against current immunotherapies.

The present study addresses these questions by examining antitumor T cell responses in murine models with T cell–specific VISTA deficiency. We demonstrate that VISTA is abundantly expressed on CD8^+^ TILs across multiple murine tumor models and various human cancer types. VISTA deficiency in tumor-specific T cells promoted their early expansion and survival. Nevertheless, this effect was transient and insufficient to control tumor growth due to T cell dysfunction driven by *trans*-VISTA on non–T cells, as global targeting of VISTA through anti-VISTA antibodies led to tumor regression. *Trans*-VISTA is highly expressed on tumor-associated myeloid cells, including MDSCs, macrophages, and DCs (14, 15), and may inhibit T cell responses through diverse mechanisms. First, VISTA acts as a *trans*-ligand and interacts with inhibitory receptors such as LRIG1 and PSGL-1 and impairs T cell expansion, survival, and effector function (7, 16). Second, VISTA expressed on myeloid progenitors promotes the differentiation and immunosuppressive function of MDSCs (15). Third, VISTA intrinsically impairs TLR-mediated signaling and suppresses the production of immune-stimulatory cytokines and chemokines such as IL-12 and CXCL9/10, thereby maintaining antiinflammatory and protumorigenic function of tumor-associated macrophages and DCs (14). Lastly, VISTA on macrophages could dampen their cytotoxicity toward cancer cells through TRAIL-mediated mechanisms (48). The multifaceted actions of myeloid-VISTA complement the cell-intrinsic effects of VISTA/LRIG1 inhibitory signaling, underscoring that global VISTA blockade is required to maximally enhance the antitumor T cell responses.

In addition to *trans*-VISTA, CTLA-4 emerged as another factor that synergizes with T cell–intrinsic VISTA to promote T cell dysfunction. Combined blockade of CTLA-4 and T cell–intrinsic VISTA optimally enhanced tumor rejection. We speculate that this synergy is mediated, in part, by the convergent effects of CTLA-4 and VISTA on TCR signaling. Supporting this notion, previous studies have shown that T cell–autonomous signaling of CTLA-4 restrains CD28-mediated costimulation, while non–T cell–autonomous effects include the promotion of Foxp3^+^ Treg cells (49–51). In parallel, T cell–intrinsic VISTA inhibits proximal TCR signaling through its receptor LRIG1 (16). Accordingly, concomitant blockade of CTLA-4 and VISTA in T cells optimally enhances costimulatory signaling and T cell activation.

Inhibitors targeting CTLA-4 have become the cornerstone for cancer immunotherapy, yet resistance remains common (52). In murine tumor models, CTLA-4 blockade alone was insufficient to induce tumor regression because T cell–intrinsic VISTA limits its therapeutic outcome. Removing T cell–intrinsic VISTA enhanced the expansion, mitochondrial function, survival, and effector function of tumor-specific CD8^+^ T cells under CTLA-4 blockade. VISTA-deficient CD8^+^ TILs upregulated genes involved in T cell activation, metabolism, and proliferative responses (e.g., IL-2/STAT5 signaling, IFN-α and IFN-γ response, mTORC1 signaling, and AKT_MTOR signaling). VISTA deficiency promoted the expansion of effector memory-like T cells, which aligns with previous findings that, in the context of CTLA-4 blockade, the expansion and improved function of effector memory-like CD8^+^ T cells are correlated with better clinical response (53–57). Moreover, VISTA-deficient CD8^+^ TILs displayed greater TCR repertoire diversity than WT T cells, with an increased number of unique clonotypes (higher richness) and a lower proportion of hyperexpanded clonotypes (more evenness). We interpret the increased TCR diversity as reflecting increased tumor recognition and reduced tumor escape, thereby promoting sustained tumor control. This finding aligns with previous studies showing that greater baseline diversity of the peripheral TCR repertoire and a higher number of expanded clones correlate with improved clinical responses to CTLA-4 inhibitor therapy (29–33).

VISTA suppresses antitumor immunity by modulating the activation of both myeloid cells and T cells (5, 14, 48, 58). The mechanistic implications and functional impact of T cell–intrinsic VISTA are reflected by a transcriptional signature enriched in VISTA-deficient CTLs. This signature comprises genes associated with IL-2/STAT5 signaling (*PIM1*, *RGS16*, and *Bhlhe40*), IFN-γ response (*BTG2*, *PIM1*, *IRF4*, and *STAT1*), mTORC1 signaling (*Bhlhe40* and *ENO1*), and T cell activation (*IFNG*, *TNF*, *CD28*, *RASGRP1*, and *FOS*). This signature indicates enhanced T cell activation and functionality and has prognostic value for predicting responses to immunotherapies. Although global VISTA inhibition can potently enhance antitumor immune responses, certain VISTA-targeted antibodies may induce myeloid cell activation and exacerbate inflammatory adverse effects (59). Our findings suggest that selectively targeting T cell–intrinsic VISTA may provide a safe and effective strategy to augment the therapeutic efficacy of CTLA-4 blockade or other T cell–stimulatory immunotherapies.

## Methods

### Sex as a biological variable.

Sex was not considered as a biological variable for studies of human data and animal models.

### Study design.

Our study examined male and female animals, and similar findings are reported for both sexes. We interrogated VISTA-dependent effects using mutant mice with T cell–specific deletion of the Vsir gene. We examined the phenotypes of WT versus VISTA-deficient CD8^+^ T cells using flow cytometry–based phenotypic studies and performed paired transcriptomic and TCR repertoire profiling at single-cell levels. We analyzed VISTA expression in human tumor-infiltrating T cells and evaluated the prognostic roles of VISTA-dependent gene signatures using publicly available databases. All preclinical experiments used sex- and age-matched mutant mice and WT littermates without investigator blinding. With the exception of scRNA-seq, all other experiments were repeated at least 2 to 3 times. All data points reflect biological replicates.

### Mice.

C57BL/6N (H-2^b^, CD45.2) and congenic C57BL/6N (H-2^b^, CD45.1) mice were purchased from Charles River Laboratories. *Vsir^–/–^* mice were originally generated on a C57BL/6N background and were obtained from Mutant Mouse Resource & Research Centers (www.mmrrc.org; stock 031656-UCD) (11, 13). OT-I TCR transgenic mice were purchased from The Jackson Laboratory (stock 003831) and bred with Thy1.1 congenic mice (stock 000406) to obtain WT OT1.Thy1.1 mice or bred with *Vsir^–/–^* mice to generate *Vsir^–/–^* OT1.CD45.2 mice. *Vsir^fl/fl^* mice were as described previously (15, 22) and were bred to CD4^cre^ on C57BL/6 background (The Jackson Laboratory, stock 022071). All animals were maintained in a specific pathogen-free facility at the Lerner Research Institute at Cleveland Clinic.

### Cell lines.

B16bl6, B16.OVA, MC38 (Sigma-Aldrich, catalog SCC172), LLC (ATCC, CRL-1642), and E0771.lmb (ATCC, CRL-3405) murine tumor cell lines were cultured in bicarbonate-free, HEPES-free RPMI-1640 supplemented with 10 mM HEPES (Life Technologies), 10% FBS, sodium pyruvate, 2 mM l-glutamine, 50 μM β-mercaptoethanol, and penicillin/streptomycin (Life Technologies). B16bl6 (female origin) cells were originally obtained from I. Fidler (The University of Texas MD Anderson Cancer Center) and were verified by in vivo growth in syngeneic mice and their expression of melanoma antigens TRP1, TRP2, and gp100 but have not been authenticated further by other methods. Cells were maintained at passages below 10 and detached using an accutase cell detachment medium (Sigma-Aldrich) for passage. Cells were examined for mycoplasma contamination every 5 to 6 months using the MycoAlert Mycoplasma Detection Kit (Lonza).

### Murine tumor models, vaccine treatments, and processing of tumor tissues.

Several tumor models were studied for VISTA expression on tumor-infiltrating CD8^+^ T cells. B16bl6 (50,000) melanoma cells or MC38 colon cancer cells (100,000) were inoculated intradermally on the flanks of female and male C57BL6 mice 6 to 8 weeks of age. E0771 breast cancer cells (50,000) were inoculated orthotopically into the mammary fat pad of female mice. LLC lung cancer cells stably expressing firefly luciferase (100,000) were injected via the tail vein. Tumor tissues were harvested when tumor size reached approximately 7–10 mm (for B16bl6, MC38, and E0771 models) or when LLC lung tumor burden was detected by in vivo bioluminescence imaging (IVIS) in the lung.

For vaccine treatment, mice bearing B16bl6 melanoma or MC38 colon cancer were treated on day 3 with a peptide vaccine mixture that consisted of CpG (ODN1826, 30 mg), R848 (50 mg), and peptides (30 mg). For the B16bl6 model, the mutated TRP2 peptide DeltaV TRP2 (residues 180–188) was used (60). For the MC38 model, neo-epitopes from Rpl18 and Adpgk proteins were used (61). All peptides were synthesized by Atlantic Peptides. The vaccine mixture was injected subcutaneously on day 3 following tumor inoculation. When indicated, tumor-bearing mice were treated subcutaneously with CTLA-4–specific mAbs (clone 9H10, Bio X Cell), 100 μg per mice, by subcutaneous injection on days 4 and 8 after tumor inoculation. Tumor size was measured with a caliper every 2–3 days. In some experiments, tumor-free mice that survived for 80 days were rechallenged with the same tumor cells, at a dose of 25,000 cells, at a distant site away from the initial tumor site.

For analyzing the phenotypes of TILs, tumor tissues were harvested on days 16–18 after vaccine treatment. In some experiments, tumor-bearing mice were injected with BrdU (1 mg per mouse) via intraperitoneal injection, 16 hours before tissue harvest and flow cytometry analysis. Single-cell suspensions were obtained after digestion with a cocktail of Liberase TL (150 μg/mL) and DNase I (120 μg/mL) (Sigma-Aldrich) for 20 minutes at 37°C followed by passing cells through 70 μm strainers. To detect intracellular cytokines, single-cell suspensions of tumor tissues (2–3 million) were stimulated in RPMI medium containing the relevant peptides (10 μg/mL), 10% FBS, 2 mmol/L l-glutamine, 50 mmol/L 2-mercaptoethanol, 1% penicillin/streptomycin, 1× monensin, and 1× Brefeldin A (BioLegend) for 6–15 hours before antibody staining.

### In vitro T cell activation analysis.

Splenic CD8^+^ T cells were purified using a negative selection kit (Miltenyi Biotec) following the manufacturer’s protocol. Purified T cells (20,000 per well) were resuspended in complete RPMI medium containing 10% FBS, 2 mmol/L l-glutamine, 50 mmol/L 2-mercaptoethanol, and 1% penicillin/streptomycin and seeded in 96-well plates that were precoated with anti-CD3 antibody (clone 2C11, 3 mg/mL). When indicated, anti–CTLA-4 antibody (clone 9H10) or isotype IgG (10 mg/mL) was added. After 48 hours of culture, T cells were harvested and analyzed by flow cytometry.

### Flow cytometry and data analysis.

For flow cytometry, antibodies specific for CD4 (GK1.5), CD8 (53-6.7), CD16/CD32 (clone 93), CD45 (30-F11), TNF-α (MP6-XT22), IFN-γ (XMG1.2), CTLA-4 (UC10-4B9), and FAS (SA367H8) were purchased from BioLegend. Caspase-8 staining was performed using the Vybrant FLICA Caspase Apoptosis Assay Kit (Thermo Fisher Scientific). Single-cell suspensions from tumor tissues and lymph nodes were stained with LIVE/Dead fixable near-IR or Aqua cell staining kits (Thermo Fisher Scientific), followed by antibody cocktails at recommended dilutions. For intracellular cytokine staining, cells were first stained with viability dye and surface lineage markers. Cells were then fixed and permeabilized using the eBioscience Intracellular Fixation & Permeabilization Buffer Set (catalog 88-8824-00) and stained using antibodies specific for intracellular cytokines. TRP2-specific dextramer (Immudex) was used to stain CD8^+^ T cells following the manufacturer’s instructions. To measure mitochondrial mass and membrane potential in CD8^+^ T cells, cells were stained with MG and MDR or with mitoSOX (Thermo Fisher Scientific) along with anti-CD8 (53-6.7) antibody. Cells were analyzed using BD LSRFortessa cell analyzer (BD Biosciences) or MACSQuant cytometer (Miltenyi Biotec). Data were analyzed using FlowJo version 9.9.6 or V10 analysis software (Tree Star).

### Adoptive T cell transfer studies.

Splenic naive Thy1.1 WT OT1 and CD45.1 *Vsir^–/–^* OT1 T cells were purified using a T cell negative selection kit (Miltenyi Biotec) according to the manufacturer’s instructions. In the pretumor transfer setting, OT1 T cells were mixed at a 1:1 ratio, validated by flow cytometry, and injected retro-orbitally (1,000 cells for each genotype) into naive CD45.2 C57BL/6 mice. Twenty-four hours after transfer, mice were inoculated with B16.OVA cells (4 × 10^6^ cells split into 4 injection sites per mouse). In the post-tumor transfer setting, mice bearing 4-day established B16OVA tumors (~3 mm in diameter) received a transfer of OT1 T cells (WT vs. *Vsir^–/–^* OT1 T cells, 2,500 each, mixed at a 1:1 ratio). On indicated days following cell transfer, tumor tissues and tumor-draining lymph nodes were harvested and examined by flow cytometry.

### Mitochondrial stress test.

Mitochondrial respiration in cultured T cells was analyzed using the Seahorse XFe96 extracellular flux analyzer (Agilent) based on protocols specified in the Seahorse Mito stress test kit (103015-100, Agilent). Cells were resuspended in XF base media (Agilent) supplemented with 25 mM glucose, 2mM l-glutamine, and 1mM sodium pyruvate, pH 7.4, without sodium bicarbonate or phenol red. OCRs were assessed in basal conditions and following the serial additions of oligomycin (1 μM), carbonyl cyanide 4-(trifluoromethoxy)phenylhydrazone (FCCP; 1.5 μM), rotenone (500 nM)/antimycin A (500 nM), and 2-deoxy-d-glucose (20 mM). All drugs were purchased from Sigma-Aldrich and resuspended in appropriate solvents prior to experiments. Probes were rehydrated 1 day prior in 200 mL MilliQ water in a 37°C non-CO_2_ incubator. Water was replaced with calibrant (Agilent) 1 hour before analysis. The 96-well Seahorse plate (Agilent) was coated with Cell-Tak (Sigma-Aldrich) for 20 minutes, aspirated, and washed 2 times with MilliQ water prior to the addition of 50 mL of T cells (2 million/mL) resuspended in base media. Plates were spun at 40*g* for 10 minutes at 37°C without breaking to stop. An additional 50 mL of prewarmed base medium was added to each well containing cells, and wells were checked under a microscope to ensure an even distribution of cells at the bottom of the wells. 100 mL of base media was added to additional wells without any cells, which served as a background control. Plates were incubated at 37°C for 30 minutes in a non-CO_2_ incubator while drugs were loaded into the ports of the assay plate. Basal respiration was calculated as the OCR measured before oligomycin minus the OCR after rotenone/antimycin A (Rot/AA) injection. Maximal respiration was determined as the OCR after FCCP treatment minus the OCR after Rot/AA. Spare respiratory capacity was determined by subtracting basal respiration from maximal respiration.

### Single-cell transcriptomic analysis and TCR-sequencing analysis.

Tumors from 5–6 *Vsir^fl/fl^* *CD4*^cre^ KO mice or *Vsir^fl/fl^* WT littermates were pooled and processed into single-cell suspensions as described above. CD4^+^ and CD8^+^ TILs were first enriched using the CD4 and CD8a Cell Isolation Kit (Miltenyi Biotec). TILs were stained with fluorescently labeled anti-CD4 and CD8 antibodies (BioLegend) in the presence of anti-CD16/32 FCR blocker (catalog 101319) on ice for 20 minutes. CD4^+^ and CD8^+^ TILs were sorted using a BD FACSAria sorter (BD Biosciences).

Viable sorted T cells were washed 3 times before being processed using the 10x Genomics Chromium Single Cell V(D)J profiling reagent kit (v1.0) according to the manufacturer’s protocols.

The scRNA libraries were prepared by the Genomics Core facility at Lerner Research Institute. The scRNA-seq gene expression and VDJ libraries were sequenced on an Illumina NovaSeq system. After sequencing, Cell Ranger version 5.0.0 was utilized to align both gene expression and VDJ libraries together using the Cell Ranger multi pipeline with the references “mouse_mm10-2020-A” and “vdj_GRCm38_alts_ensembl-5.0.0.” After alignment, the WT sample had 303 million reads for gene expression and 117,000 reads for VDJ, corresponding to 10,337 cells with a median gene per cell count of 1,913. The VISTA-KO sample similarly had 286 million reads for gene expression and 52,000 reads for VDJ, corresponding to 10,085 cells with a median gene per cell count of 1,877.

After data alignment, Seurat version 4.0 in R version 4.1 was used for all downstream analysis and data integration. For each cell, the percentage of mitochondrial and ribosomal genes was calculated and used to filter out dead/dying cells. Cells were included for subsequent analysis if they contained at least 600 features of RNA and less than 30% mitochondrial content. After normalization, samples were integrated using the Harmony R package (https://portals.broadinstitute.org/harmony/). Clusters were subsequently generated using Seurat functions and were manually identified using top marker genes and using the FindAllMarkers function in Seurat with default parameters. VDJ analysis was performed using scRepertoire 2.0.7 (https://github.com/BorchLab/scRepertoire) (62). Using scRepertoire, the VDJ data from Cell Ranger was integrated with Seurat for comprehensive analysis of the clonal changes across populations and between samples.

To generate the VISTA^deficient^ CTL gene signature module, we identified statistically significant (*P* adjust < 0.05) upregulated genes in the VISTA-KO CD8^+^ TILs and mapped them to their human orthologs using the babelgene R package (R package version 22.9, https://igordot.github.io/babelgene/). We calculated the difference in the fraction of cells expressing each gene between VISTA-deficient and WT TILs. We then ranked all genes by this difference, from highest to lowest, and selected the top 40 to define the gene signature.

To assess the expression of this signature in CD8^+^ TILs, signature scores were computed with the AddModuleScore() function using the Seurat R package. To examine *Ctla4* gene expression across CTL subsets, we stratified cells into “high” and “low” groups based on the median signature score and visualized *Ctla4* expression using Seurat’s DotPlot() function and the ggplot2 R package.

To evaluate the prognostic value of this CTL signature, we assessed its expression using the GSVA R package (63) by performing the single-sample GSEA across publicly available bulk RNA-seq datasets (46, 47). Raw expression values of the signature genes were used as input. Samples were stratified into high and low expression groups based on the median enrichment score. Signature scores were visualized alongside clinical response and survival outcomes using the ggplot2, survival (R package version 3.8-3, https://CRAN.R-project.org/package=survival), and survminer R packages (R package version 0.5.0, https://rpkgs.datanovia.com/survminer/index.html) (64, 65).

### Statistics.

All graphs and statistical analysis were generated using Prism 8 (GraphPad Software). Unpaired 2-tailed *t* tests were used for comparing the phenotypes between 2 independent groups. One-way ANOVA was used to compare the phenotypes between 3 or more independent groups. A 2-way ANOVA analysis with a Šidák’s multiple-comparison test was used to compare tumor growth between groups. Survival differences were assessed using Kaplan-Meier curves and analyzed by log-rank testing. All data are presented as mean ± SEM. A *P* value less than 0.05 was considered statistically significant. Statistical information about specific experiments can be found in the figure legends.

### Study approval.

All animal protocols were approved by the IACUC of Lerner Research Institute at the Cleveland Clinic Foundation. All methods were performed in accordance with the relevant guidelines and regulations. Studies of human specimens were approved by the IRBs of the Cleveland Clinic, University Hospitals Cleveland, and Case Western Reserve University School of Medicine.

### Data availability.

The scRNA-seq data and scTCR-seq data have been deposited to the National Center for Biotechnology Information Gene Expression Omnibus (GSE290660). The code used for data analysis in this manuscript will be provided upon request. All other data are presented in the main figures or the supplemental materials. All mice are either commercially available or will be made available under a material transfer agreement. Requests for reagents should be directed to the corresponding author (LLW). Values for all data points in graphs are reported in the Supporting Data Values file.

## Author contributions

Conceptualization: LLW. Methodology: CG, ED, PBP, DR, HMT, AZ, PEG, SP, KZ, IJ, BM, SEW, TAC, TA, and LLW. Materials and reagents: RR, ZAH, AT, NLS, SA, and LLW. Experimental design, data acquisition, and data analysis: CG, ED, PBP, DR, HMT, AZ, PEG, SP, KZ, IJ, SA, TA, and LLW. Funding: LLW and CG. Supervision: TA and LLW. Writing and editing: CG, SP, PBP, TA, and LLW. CG, ED, and PBP share the first authorship due to their equally significant contributions to experimental design and data analysis. Their authorship position is determined alphabetically.

## Funding support

This work is the result of NIH funding, in whole or in part, and is subject to the NIH Public Access Policy. Through acceptance of this federal funding, the NIH has been given a right to make the work publicly available in PubMed Central.

- NIH grants R01CA164225, R01CA223804 and R21CA258618 (to LLW).
- American Cancer Society Research Scholar grant RSG-18-045-01-LIB (to LLW).
- Department of Defense CDMRP Melanoma Research Program ME210229 (to LLW).
- Department of Defense Breast Cancer Research Program BC240100 (to LLW).
- NIH grant F31CA257276 (to CG).

## Supplementary Material

Supplemental data

Unedited blot and gel images

Supporting data values

## Version 1 03/16/2026

Electronic publication
