# Supplementary material for: T cell–intrinsic VISTA expression promotes resistance to CTLA-4 blockade by restricting CD8+ T cell responses
Source: J Clin Invest. 2026 Mar 16;136(6):e195668. doi: 10.1172/JCI195668 (PMC12987620; doi:10.1172/JCI195668)
Supplement: Supplemental data [file jci-136-195668-s154.pdf]

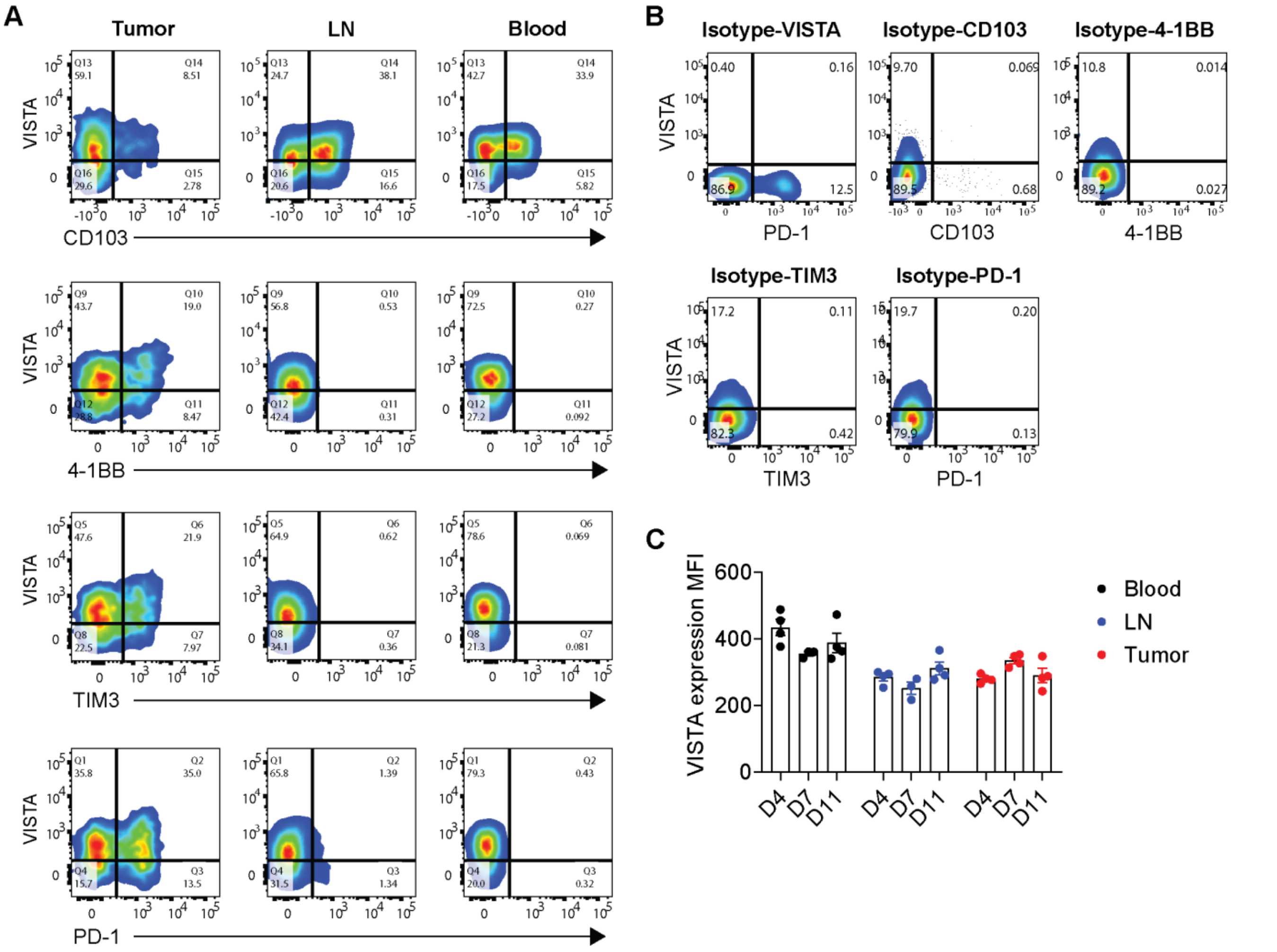

Supplemental Figure S1. VISTA expression on CD8⁺ T cells from B16-OVA tumor-bearing mice. Tumor tissues, tumor-draining LN, and blood from mice bearing B16-OVA tumors were harvested on day 4, day 7, and day 11 post tumor inoculation. T cells were stained by antibodies specific for VISTA, CD103, 4-1BB, TIM3, and PD-1, and analyzed by flow cytometry. (**A**) Representative flow plots show the co-expression of VISTA and other surface markers. (**B**) Isotype control staining for each protein. (**C**) VISTA expression on $CD8^+$ T cells was quantified by mean fluorescence intensity (MFI) and shown here.

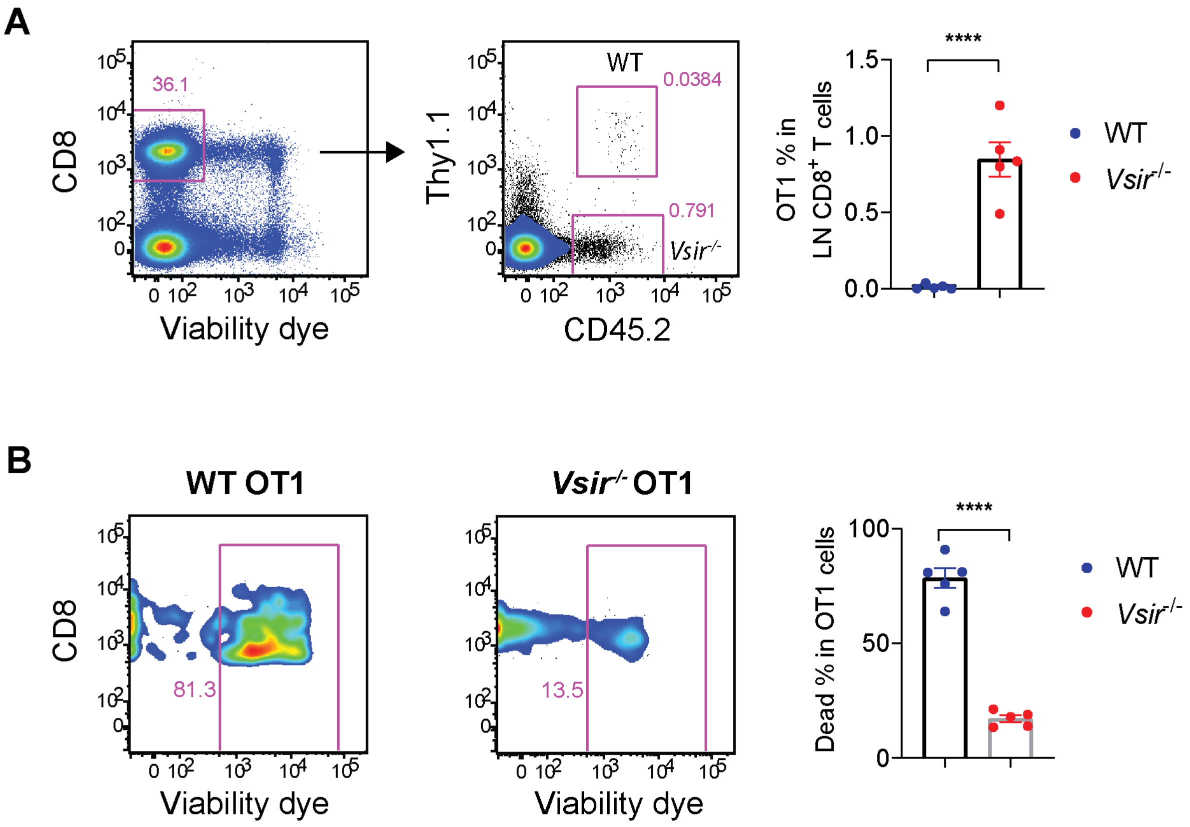

Supplemental Figure S2. T cell-intrinsic VISTA deficiency augmented the survival and accumulation of tumor-specific T cells in tumor-draining lymph nodes.

Congenically marked WT OT1 (Thy1.1^{+}) and *Vsir*$^{-/-}$ OT1 (CD45.2^{+}) were mixed at 1:1 ratio (2,500 cell each genotype) and adoptively co-transferred into C57bl/6 mice bearing 4-day established B16.OVA tumors. On day 11 post transfer, tumor-draining lymph nodes were harvested and analyzed by flow cytometry. (**A**) Representative flow cytometry plots depict the gating strategy. Percentages of WT and *Vsir*$^{-/-}$ OT1 T cells among total CD8^{+} T cells were quantified and presented. (**B**) Representative flow cytometry plots depict the detection of dead OT-I cells. Percentages of dead cells were summarized. N=5 in both panels.

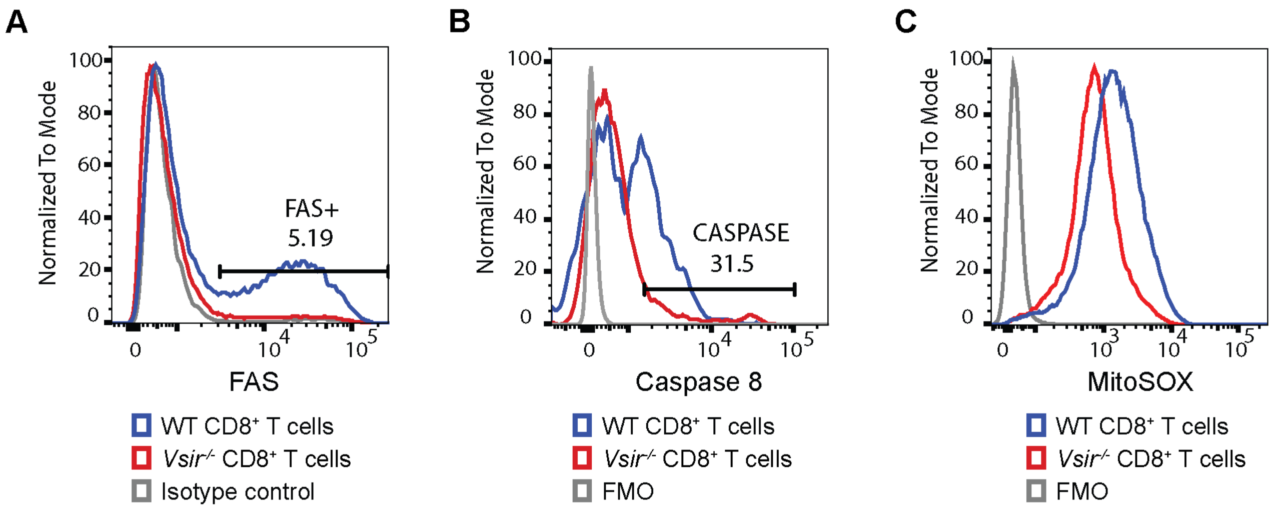

Supplemental Figure S3. Gating strategy for assessing the expression of FAS, activated Caspase-8, and mitochondrial ROS in CD8^{+} T cells. Splenic WT and *Vsir*$^{-/-}$ CD8^{+} T cells were purified and stimulated by plate-bound anti-CD3 (3 μg/mL) for 48 hours. Expression of FAS (**A**), activated Caspase-8 (**B**), and mitochondrial ROS levels (**C**) were analyzed by flow cytometry. Representative plots were shown.

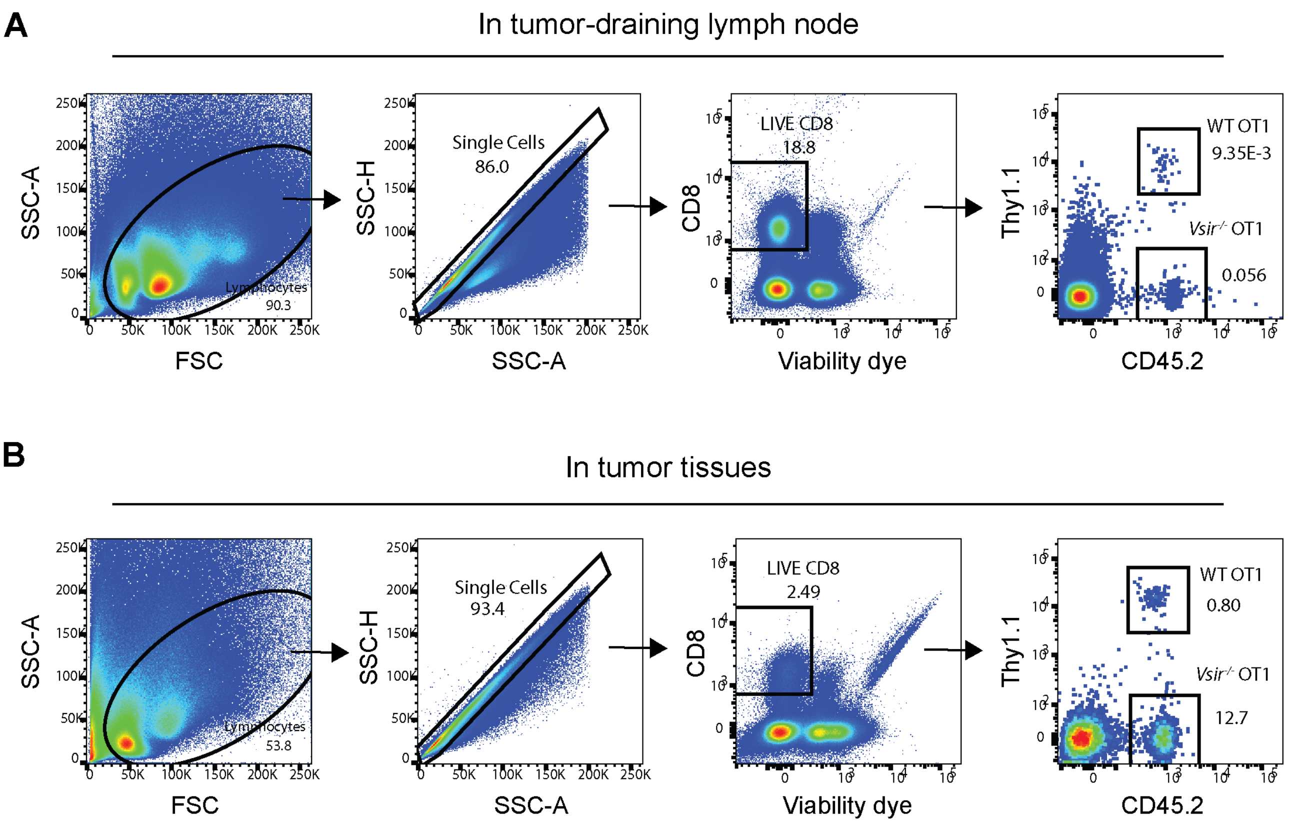

Supplemental Figure S4. Analysis of OT1 T cells transferred into recipient mice before tumor inoculation. Congenically marked WT OT1 (Thy1.1$^+$CD45.2$^+$) and *Vsir*$^{-/-}$ OT1 (CD45.2$^+$) were mixed at 1:1 ratio (1,000 cell per genotype) and adoptively co-transferred into naive C57bl/6 mice one day prior to B16.OVA tumor inoculation. On day 12 post transfer, tumor-draining lymph nodes and tumor tissues were harvested and analyzed by flow cytometry. Representative flow cytometry plots demonstrate the gating strategy for WT vs *Vsir*$^{-/-}$ OT1 T cells in tumor-draining lymph node (**A**) and in tumor tissues (**B**). Representative results from 2 different experiments were shown.

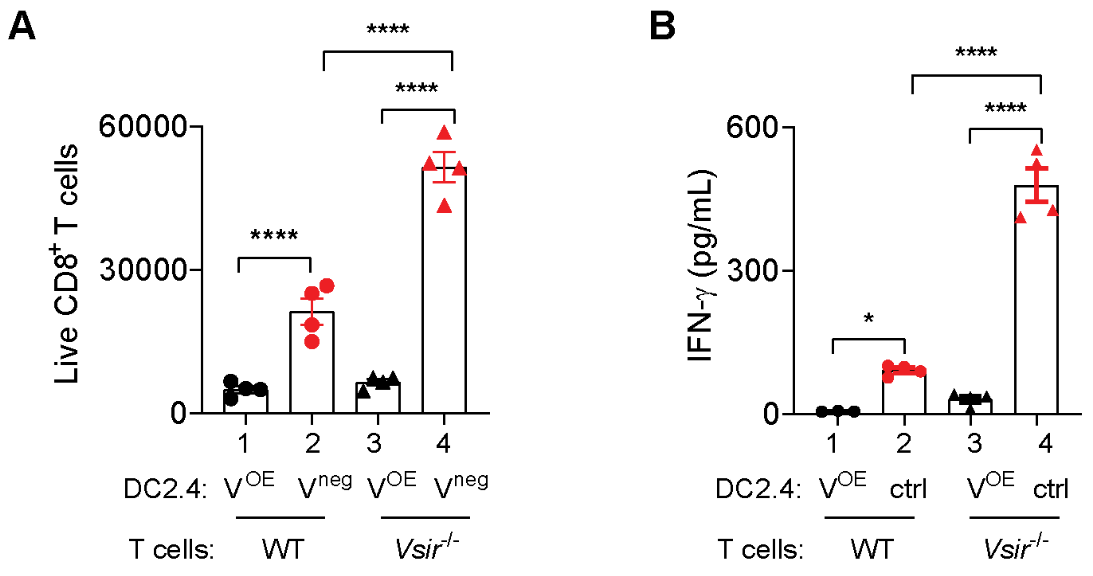

Supplemental Figure S5. The role of Trans- vs cis- VISTA in suppressing T cell activation. WT and *Vsir*$^{-/-}$ OT1 T cells were cocultured with VISTA-expressing (V^{OE}) or parental (V^{neg}) DC2.4 cells and ovalbumin peptides. Live OT1 T cells were enumerated by flow cytometry after 96 hours of culture. Culture supernatant was collected at 48 hours. Secreted IFN-γ was measured by ELISA. N = 4. * $P<0.05$, **** $P<0.0001$.

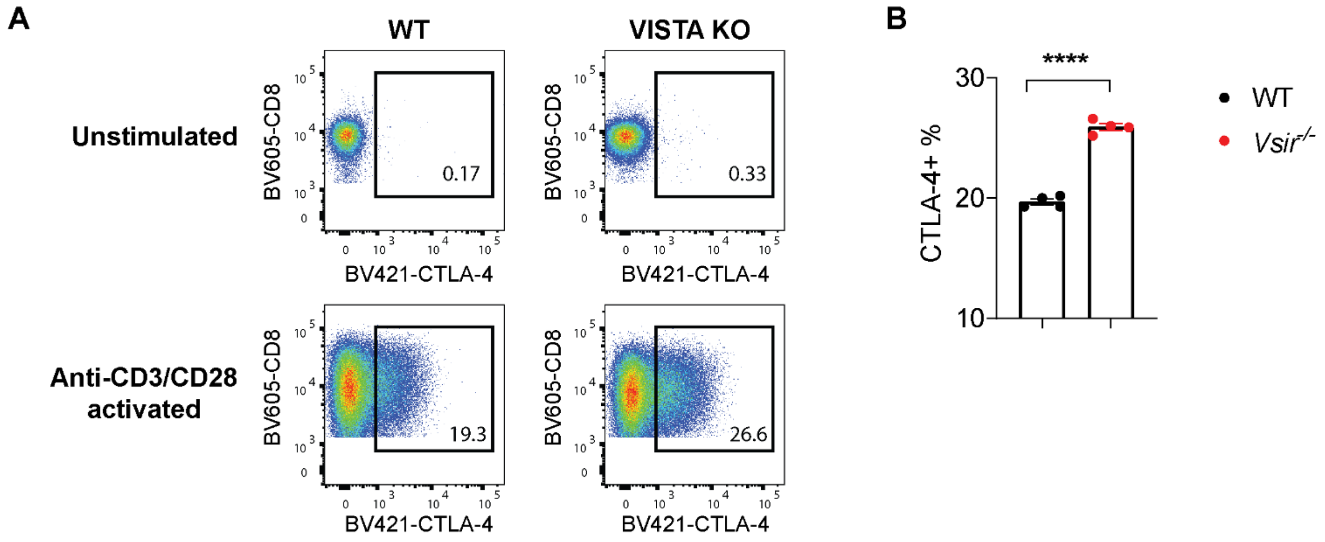

Supplemental Figure S6. CTLA-4 expression was upregulated in VISTA-deficient CD8$^+$ T cells. Naïve WT and *Vsir*$^{-/-}$ CD8$^+$ T cells were purified from splenocytes and stimulated with immobilized anti-CD3 (2 μg/mL) and CD28 (2 μg/mL) for 24 hrs. Cells were harvested and stained with anti-CTLA-4 specific antibody and analyzed by flow cytometry. (**A**) Representative flow plots showing CTLA-4 expression in unstimulated and activated CD8$^+$ T cells. (**B**) The percentages of cells expressing CTLA-4 were summarized. N=4. ****P<0.0001.

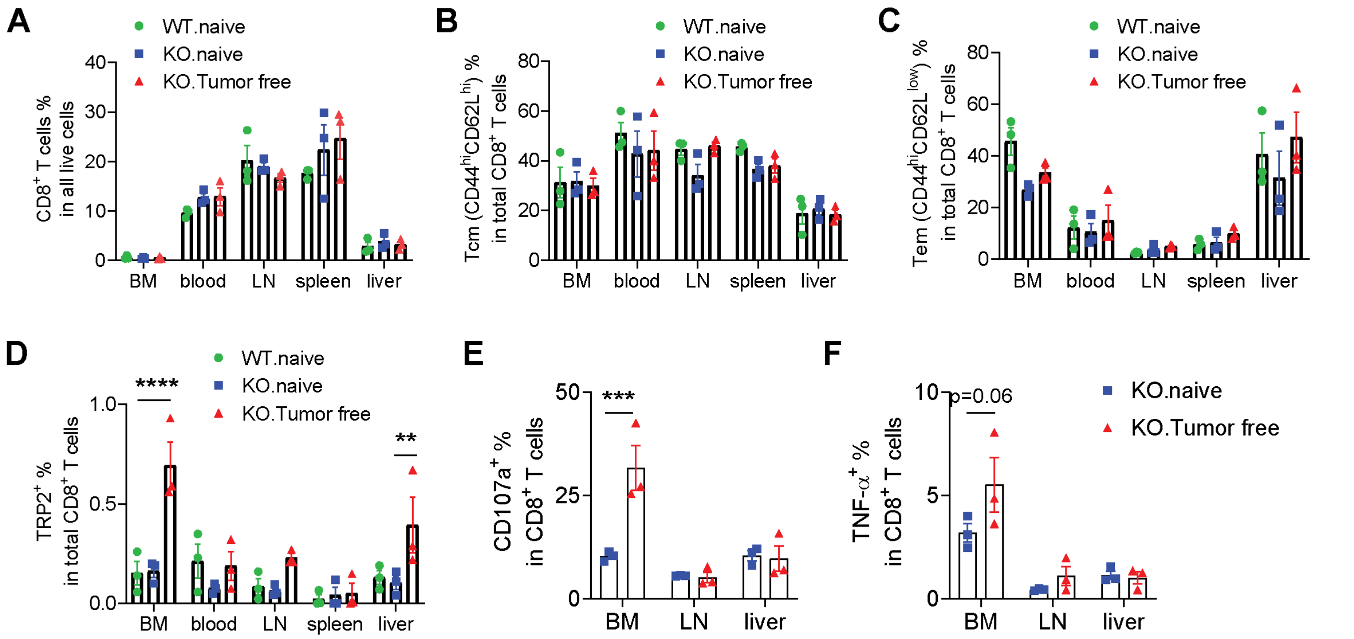

Supplemental Figure S7. Analysis of the number and phenotype of CD8$^+$ T cells in tumor-free VISTA KO mice. T cell-specific VISTA KO mice (*Vsir$^{fl/fl}$ CD4^{Cre}*) that remained tumor-free for > 60 days were analyzed. Naïve WT and VISTA KO mice were analyzed as parallel controls. Spleen, draining lymph node (LN), liver, bone marrow (BM), and blood were harvested for flow cytometry analysis. Shown were the frequencies of CD8$^+$ T cells among all live cells (**A**), the frequencies of CD44^{hi} D62L^{hi} central memory cells (Tcm) (**B**), and CD44^{hi} D62L^{low} effector memory cells (Tem) (**C**) within CD8$^+$ T cells. TRP2-specific CD8$^+$ T cells were detected using TRP2-specific dextramer and shown in **D**. To assess the functional responses of TRP2-specific CD8$^+$ T cells, T cells from BM, LN, and liver were activated *ex vivo* in the presence of TRP2 peptides for 16 hrs. The expression of CD017a and TNF-α was analyzed by flow cytometry and shown in **E-F**. N =3 in all panels. P value was assessed using two-way ANOVA. ***P<0.001, ****P<0.0001.

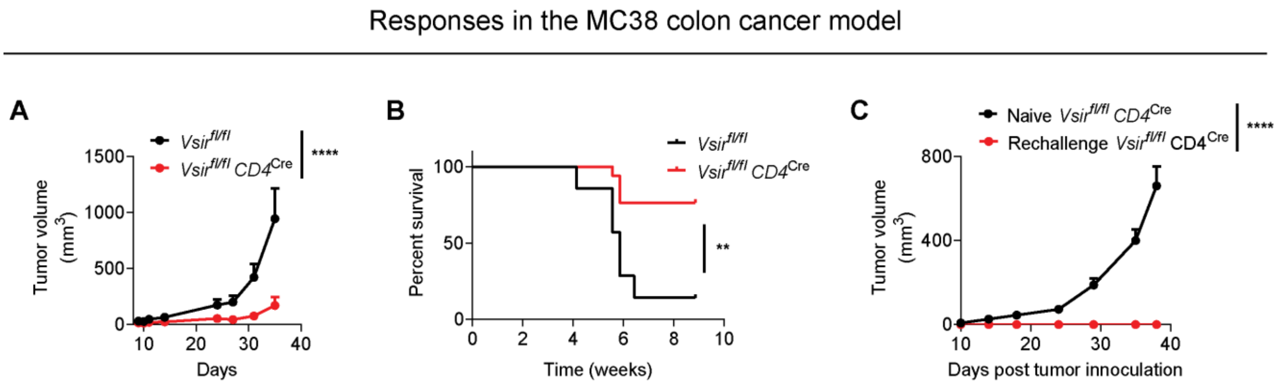

Supplemental Figure S8. CTLA-4 blockade combined with T cell-intrinsic VISTA deficiency effectively control tumor growth in MC38 colon cancer model. **(A)** T cell-specific VISTA KO mice (*Vsir*$^{fl/fl}$ *CD4*^{Cre}) and WT littermates were inoculated with 50,000 MC38 colon cancer cells on day 0. On day +3, mice received a soluble peptide vaccine formulated with CpG, R848, and peptides. Anti-CTLA-4 antibodies (clone 9H10, 150 µg per mouse) were administered on days +4 and +7. Tumor growth was monitored by a caliper every 3-4 days and shown. (**B**) Tumor-free survival of mice was monitored for up to 9 weeks post tumor inoculation. (**C**) VISTA KO mice that remained tumor free for 9 weeks were rechallenged with the same MC38 tumor cells (50,000) at a distant site and monitored for tumor progression and survival. Error bars represent SEM. For panels A-B, N=7 (*Vsir*$^{fl/fl}$), 17 (*Vsir*$^{fl/fl}$*CD4*^{Cre}). For panels C, N= 7 (naïve *Vsir*$^{fl/fl}$*CD4*^{Cre}), 11 (tumor-free surviving *Vsir*$^{fl/fl}$*CD4*^{Cre}). **$P<0.01$, ****$P<0.0001$. P value was assessed using Student's T test between two groups. Representative results of 2 independent experiments were shown.

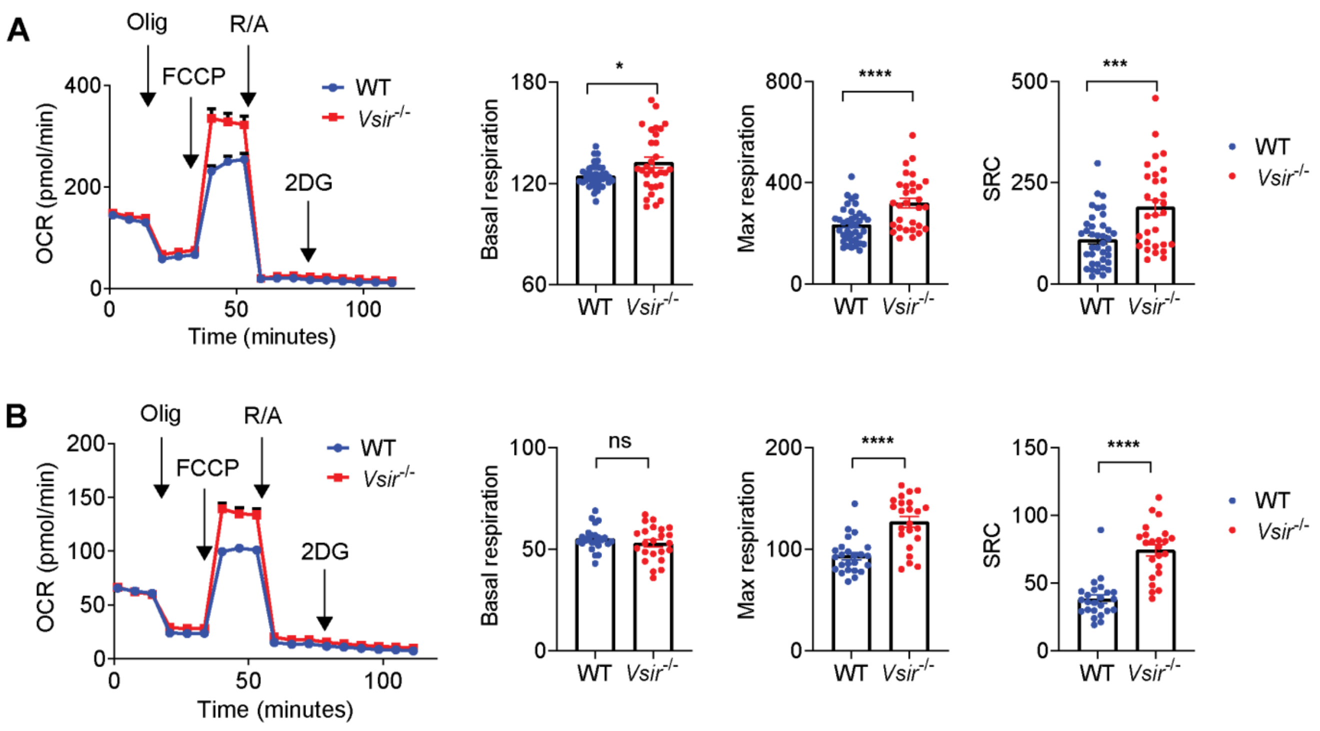

Supplemental Figure S9. Enhanced mitochondrial function in VISTA-deficient T cells upon acute activation and after chronic stimulation. **(A)** Naïve WT and *Vsir*$^{-/-}$ OT1 T cells were isolated from the mice spleen and activated in vitro by immobilized anti-CD3 (0.5 µg/mL) and CD28 (0.25 µg/mL) antibodies. After 48 hours, cells were harvested and mitochondrial respiration was assessed using the Agilent Seahorse Mito Stress Test on the Seahorse XFe96 Extracellular Flux Analyzer. Shown are OCR levels at baseline and after treatment with Oligomycin (Olig), FCCP, rotenone/antimycin (R/A), and 2DG. The basal respiration, maximal respiration, and spare respiratory capacity (SRC) were calculated as described in the Methods and presented. (**B**) WT and *Vsir*$^{-/-}$ OT1 T cells were stimulated twice with anti-CD3 and CD28. At 48 hours after second stimulation, cells were harvested and analyzed as in A. Error bars represent SEM. N=44 in panel A. N=44 in panel B. *P<0.05, ***P<0.001, ****P<0.0001. P value was assessed using Student's T test between two groups. Representative results of 2 independent experiments were shown.

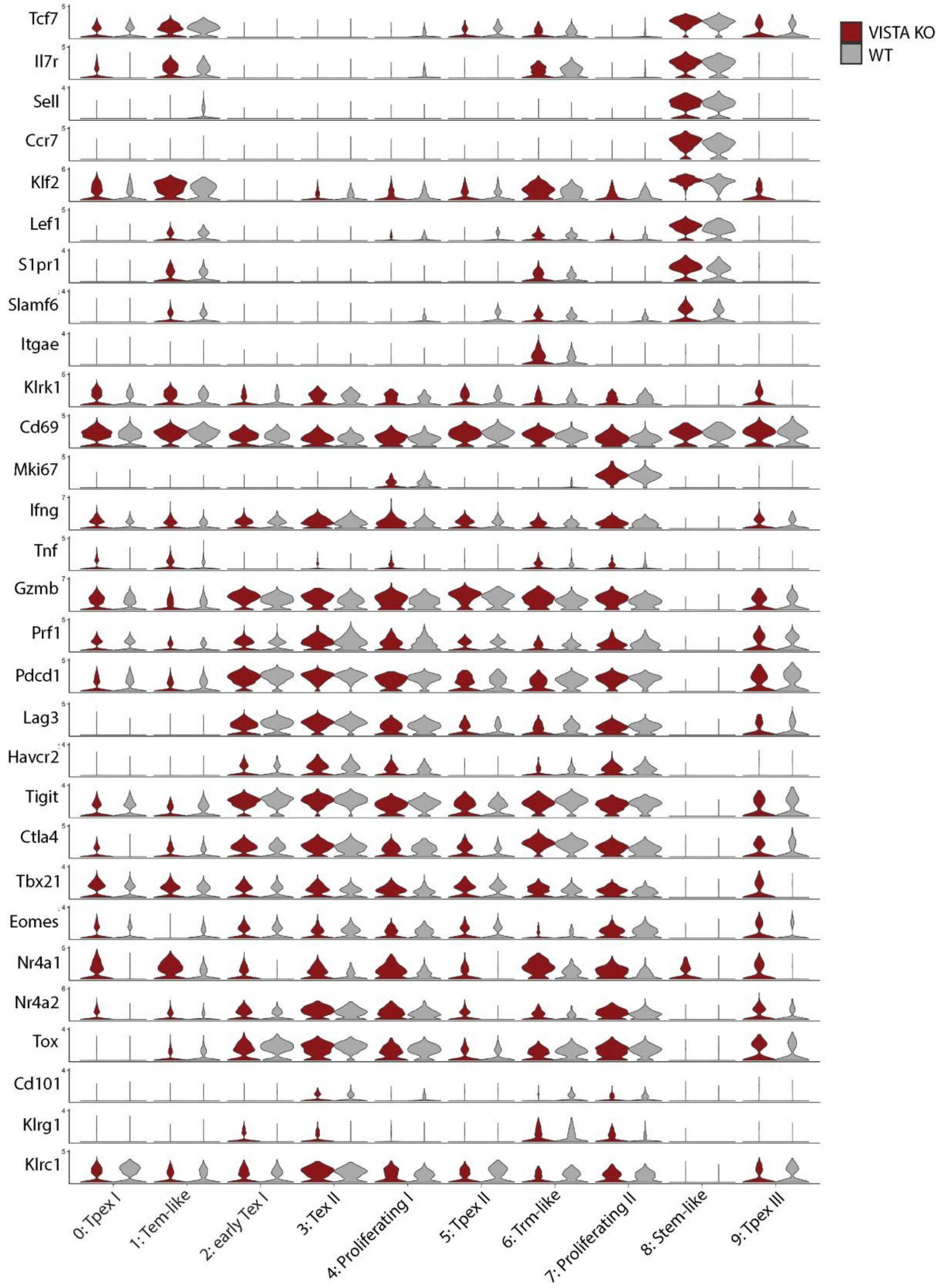

Supplemental Figure S10. Single-cell transcriptomic analysis of CD8$^+$ TILs. This is extended data related to Figure 5. Violin plots show the expression of marker genes that are associated with the differentiation and activation states of CD8$^+$ TILs.

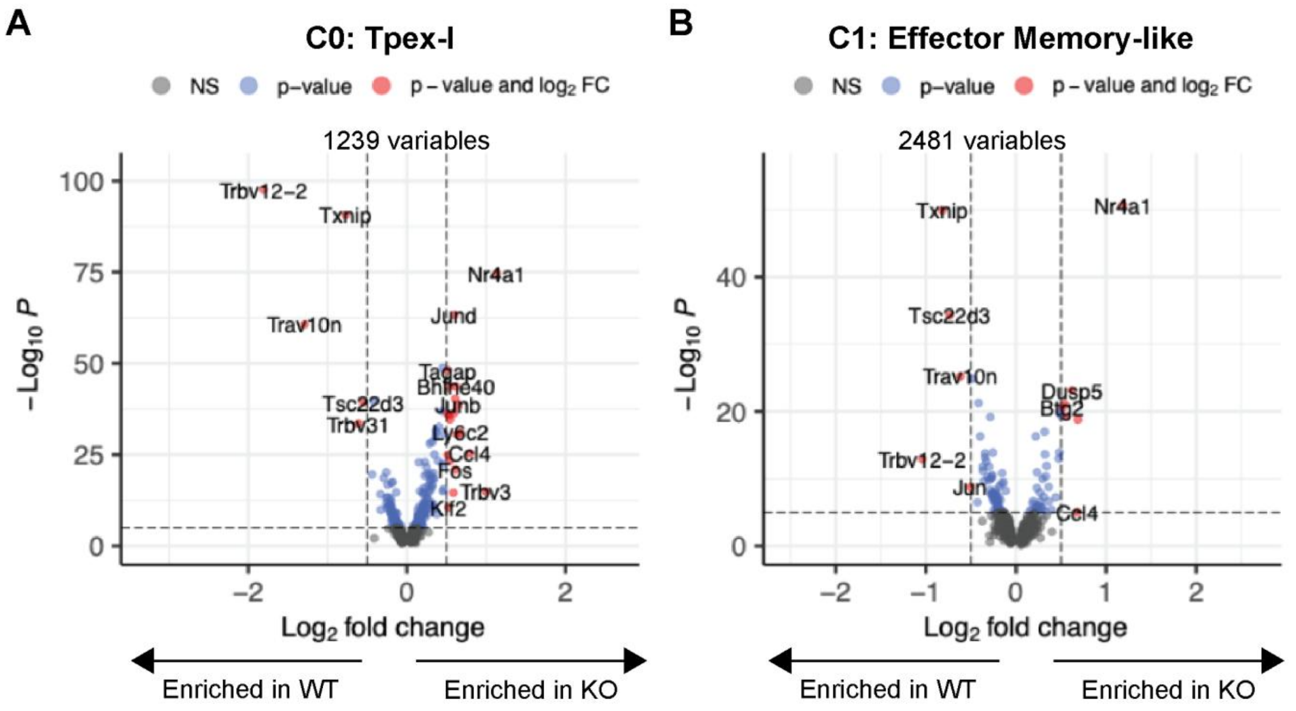

Supplemental Figure S11. Volcano plots display differentially expressed genes in the Tpex-I (C0) and Effector Memory like (C1) subsets of murine CD8^{+} TILs.

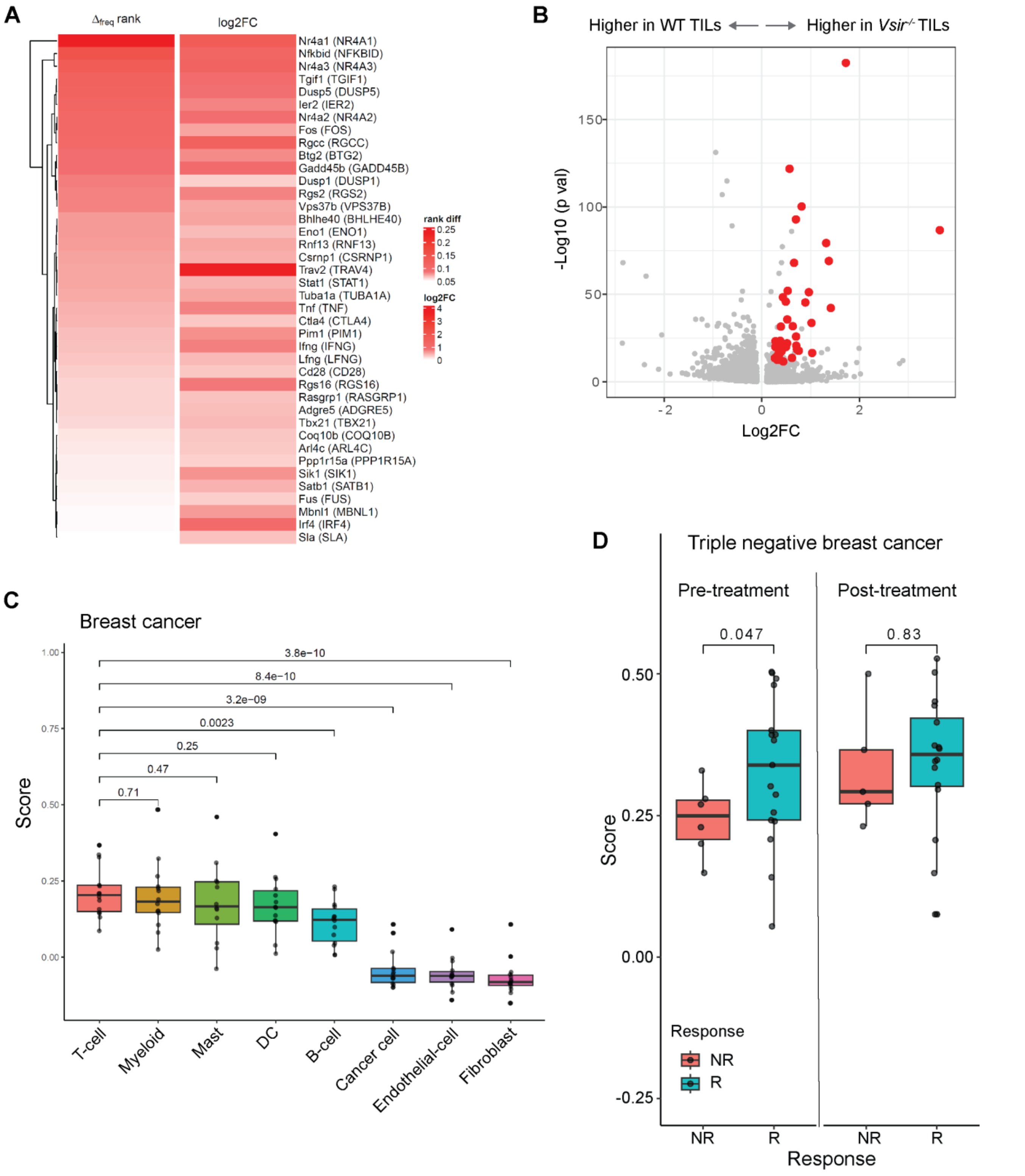

Supplemental Figure S12. The generation of a gene signature enriched in VISTA-deficient CD8$^+$ TILs and its expression in human breast cancer. A 40-gene signature enriched in VISTA-deficient murine CD8$^+$ TILs was identified. (**A**) Heatmap depicting gene rankings based on expression frequency

and fold change analyses. (**B**) Volcano plot displaying the log fold changes of ranked genes. (**C**) Expression of the CTL signature was assessed across breast cancer-associated cell populations using a public single-cell RNA-seq dataset. (**D**) Expression of the signature was assessed in $CD8^+$ TILs from triple-negative breast cancer.

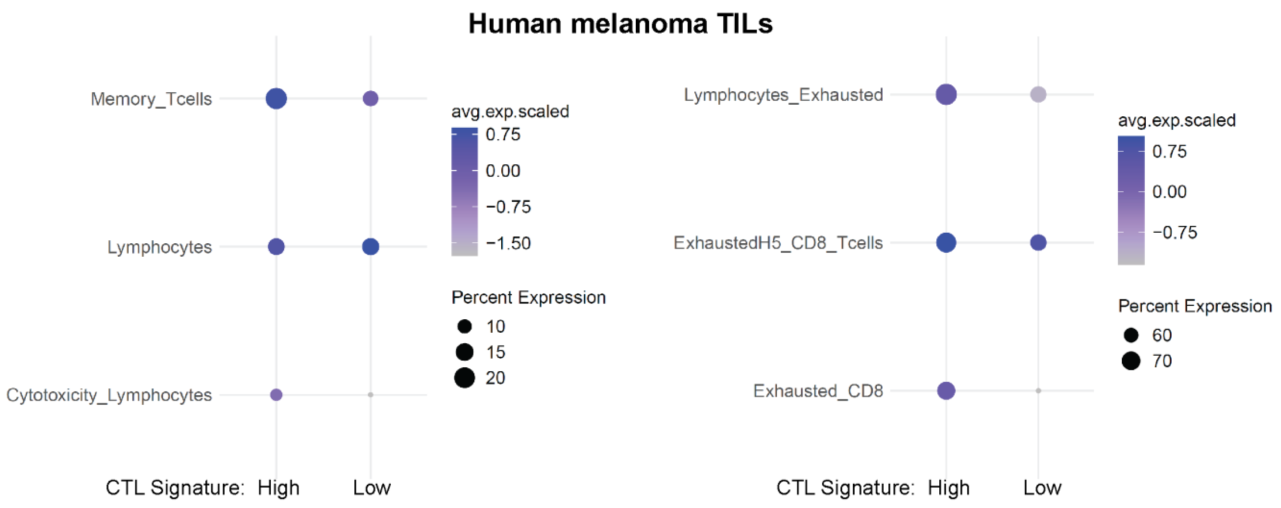

Supplemental Figure S13. Subsets of $CD8^+$ TILs from melanoma tissues were stratified into "High" and "Low" groups based on the median signature score. *Ctla4* expression was analyzed and visualized using dot plots.
