# Supplementary figures and images for: T cell–intrinsic VISTA expression promotes resistance to CTLA-4 blockade by restricting CD8+ T cell responses
Source: J Clin Invest. 2026 Mar 16;136(6):e195668. doi: 10.1172/JCI195668 (PMC12987620; doi:10.1172/JCI195668)

Bcl-XL band: 30kDA

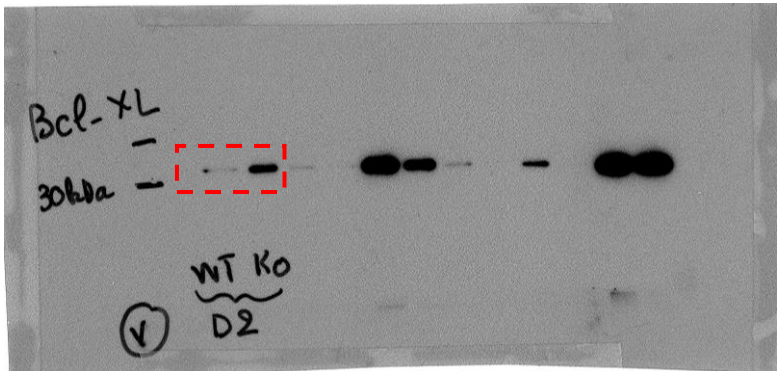

Actin band: 43kDA

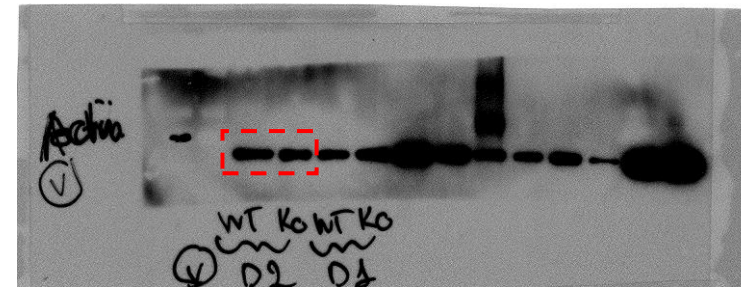

Supplement: Unedited blot and gel images [file jci-136-195668-s155.pdf]
